# Intelligent Thinning Decision for Strawberry Quality Optimization: A Lightweight Peduncle–Fruit Topological Relationship Perception Method

**DOI:** 10.3390/foods15183223

**Published:** 2026-09-11

**Authors:** Hongjun Luo, Zaosong Li, Fuguo Xie, Ya Yue, Yun He, Gao Quan

**Affiliations:** 1 College of Big Data, Yunnan Agricultural University, Kunming 650201, China; 2024240722@stu.ynau.edu.cn (H.L.); 2024240740@stu.ynau.edu.cn (Z.L.); 2021250128@stu.ynau.edu.cn (F.X.); 2 Key Laboratory for Crop Production and Intelligent Agriculture of Yunnan Province, Yunnan Agricultural University, Kunming 650201, China; 2014005@ynau.edu.cn; 3 College of Economics and Management, Yunnan Agricultural University, Kunming 650201, China

**Keywords:** strawberry thinning, food safety, hierarchical segmentation, edge computing, peduncle–fruit topological association

## Abstract

Fruit thinning concentrates nutrients and serves as an essential procedure for cultivating high-quality strawberries with exceptional palatability. It is established that controlling the number of fruits per peduncle to three to five significantly increases the proportion of large fruits and enhances sweetness while effectively reducing the risks of disease infection. Consequently, pesticide application is minimized, thereby ensuring food safety and improving overall product quality. Although precise execution baselines are a prerequisite for automated thinning, current research is largely restricted to isolated fruit recognition, leaving the direct detection of peduncle–fruit topological associations under complex occlusions unaddressed. To address this gap, a lightweight, three-stage (“coarse-to-fine”) detection method tailored for automated thinning is proposed. The pipeline comprises global localization, local cropping with background suppression, and fine secondary inference, which effectively mitigates environmental interference. To support this investigation, a dedicated dataset comprising 1131 high-quality images was constructed. For efficient edge deployment, YOLOv11n was adopted as the baseline architecture, integrated with structural Re-parameterized Convolution (RepConv), Coordinate Attention (CoordAtt), and a dynamic re-weighting loss. This configuration ensures robust feature extraction of slender peduncles with an extremely low parameter overhead. The experimental results demonstrate that with only 2.77 M parameters, the proposed model achieves an mAP@0.5 of 75.73% and an F1-score of 73.85%. Notably, the average false positive (FP) detections per image in complex scenarios were significantly reduced from 1.53 to 0.23. Following deployment on a Jetson Orin Nano Super edge device utilizing TensorRT and FP16 quantization, the end-to-end system inference speed stabilized at 15.58 frames per second (FPS) with near-lossless precision. Ultimately, this method provides a reliable technical foundation for automated thinning decisions, facilitates sustainable greenhouse management, and secures the supply of high-quality food from the source.

## 1. Introduction

High-quality strawberries are fundamentally characterized by a high proportion of large fruits, elevated sugar content, and superior sensory attributes; these objectives are predominantly achieved through scientifically regulated fruit thinning and harvesting operations. The optimization of nutrient distribution is realized by strictly controlling the number of retained fruits. Through this concentrated nutritional supply, robust fruit development is promoted, directly enhancing both the sweetness and palatability of the strawberries. Furthermore, the spatial topological distribution of fruits is optimized through rational thinning practices, whereby disease incidence within dense clusters is significantly reduced. Consequently, pesticide reliance is minimized during the growth period, ensuring food safety and economic viability from the source while enhancing overall sensory quality. Compared with the unthinned natural control group, scientific thinning (e.g., retaining five fruits per inflorescence) has been demonstrated to significantly elevate the average proportion of large fruits from 85.53% to 97.37%, while the average single-fruit weight during the late harvesting period is increased from 14.56 g to 19.36 g [1]. In traditional agricultural management, these operations are executed predominantly through manual visual judgment and physical cutting, which are inherently labor-intensive, costly, and characterized by a low degree of operational standardization. With the exacerbation of global agricultural labor shortages, exorbitant labor costs have severely restricted the large-scale, intensive development of the strawberry industry [2]. Research indicates that when the number of retained fruits exceeds four, yield is no longer significantly increased; conversely, when six fruits are retained, both yield and quality decline. Under greenhouse conditions, it has been established that the optimal fruit load consists of one primary fruit and two secondary fruits [3]. To satisfy these stringent requirements regarding quantity and hierarchy, automated systems must precisely recognize and associate the peduncle connection relationships. Therefore, the investigation of rapid, accurate, and practical automated perception methods for strawberry organ hierarchy is of paramount importance for the realization of large-scale agricultural robotic decision-making systems and automated collaborative operations [4].

Although explicit identification of organ-level topological relationships is a prerequisite for automated fruit thinning, early machine vision studies were primarily limited to basic fruit detection and counting without addressing the precise peduncle–fruit associations required for robotic operations. Conventional image processing techniques [5] and representative two-stage deep learning networks (e.g., Mask R-CNN) [6,7] have been utilized for agricultural segmentation; however, they exhibit limited robustness or incur substantial computational overhead when confronted with overlapping targets and severe occlusion caused by dense foliage [8,9]. To address target occlusion, recent research has gradually shifted toward organ-level perception. For instance, Yang et al. developed a deep learning-based detection framework incorporating a self-attention mechanism to estimate fruit numbers and localize peduncle regions, thereby improving detection accuracy in densely populated scenes [10].

To address severe target occlusion and mutual overlapping in densely clustered crops while meeting the stringent low-latency requirements of practical robotic deployment, various advanced deep learning architectures and lightweight one-stage detectors have been actively investigated [11,12,13,14,15]. In particular, lightweight detectors and optimized YOLO variants (such as YOLOv4 and YOLOv5) have demonstrated considerable potential in suppressing complex background interference and accurately distinguishing closely packed fruits [16,17,18,19]. While recent studies have improved YOLO architectures for video-based crop counting [20] and peduncle keypoint detection [21], extracting complex topological structures under severe occlusion directly on resource-constrained mobile edge platforms remains a significant challenge [22].

**Figure 1:**
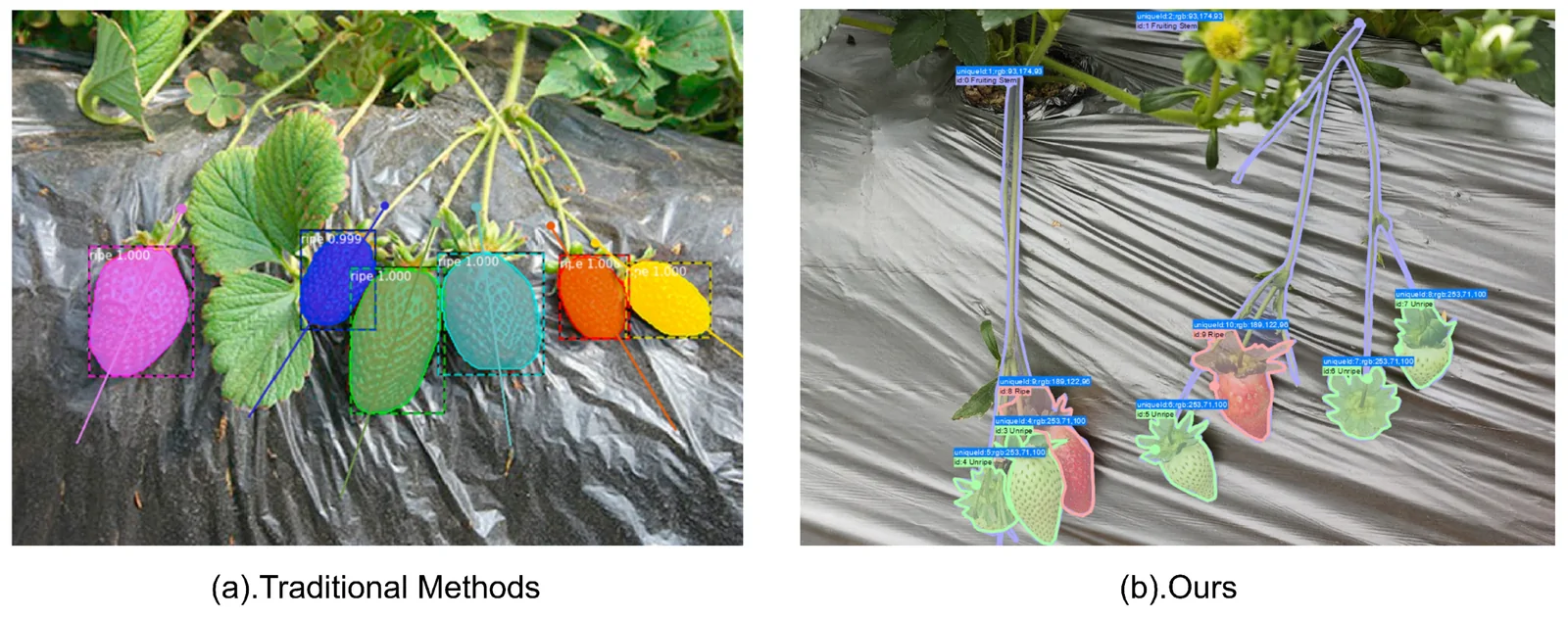
Comparison of the results of traditional single-fruit segmentation and the peduncle topology-based segmentation method proposed in this paper.

In summary, as illustrated by the visual comparison in Figure 1, existing machine vision studies on agricultural crop perception have primarily focused on improving the detection accuracy and counting performance of individual fruits (Figure 1a) [23], while little attention has been devoted to the analysis of plant organ-level topological structures, particularly the direct identification of peduncle–fruit associations that are essential for automated fruit thinning. To bridge this research gap and mitigate the severe occlusion commonly encountered in complex agricultural environments, the focus of this study was extended from individual fruit detection to the extraction of plant organ-level topological relationships. Accordingly, a single-model, multi-stage cascaded inference framework was proposed. The framework first localizes potential fruit cluster regions through global detection, followed by local cropping and background suppression to perform fine-grained secondary inference. This staged strategy effectively suppresses interference from complex backgrounds and accurately establishes topological correspondences between fruits and their associated peduncles, thereby enabling precise instance segmentation of strawberry peduncles and fruits across different developmental stages [24].

To support the efficient execution of the proposed cascaded framework, the YOLOv11n baseline was further optimized for lightweight deployment. By integrating Coordinate Attention (CoordAtt) [25] and Structural Re-parameterized Convolution (RepConv) [26], the network was enabled to focus more effectively on local discriminative features while maintaining low computational complexity. In addition, a dynamic re-weighting strategy based on the Focal Tversky Loss was incorporated to alleviate the boundary segmentation challenges associated with extremely imbalanced targets, particularly slender peduncles. Consequently, a cost-effective and highly reliable perception solution was established for automated fruit thinning in smart agriculture systems [27].

The main contributions of this study are summarized as follows:

(1) A coarse-to-fine, three-stage hierarchical perception pipeline was proposed. A cascaded strategy consisting of global localization, local cropping with background suppression, and fine-grained secondary inference was engineered to effectively isolate environmental background interference. The proposed framework substantially reduced the per-image false positive (FP) rate while establishing accurate peduncle–fruit topological associations, thereby significantly mitigating the risk of erroneous automated decision-making.

(2) A dedicated benchmark dataset focusing on strawberry organ-level topology was constructed. Multi-view images were collected across both elevated substrate and ground-based greenhouse cultivation facilities, and a high-quality dataset was established using domain-specific data augmentation. Fine-grained polygon mask annotations were provided to compensate for the deficiency of slender peduncle annotations in existing public datasets, thereby considerably enhancing the model generalization capability.

(3) A favorable trade-off between model lightweightness and segmentation accuracy was achieved. Built upon the YOLOv11n architecture, RepConv modules were integrated to enhance feature extraction, while the CoordAtt mechanism and the Focal Tversky Loss were jointly adopted to strengthen the localization of slender peduncles and suppress long-tail missed detections. Consequently, superior detection performance was maintained with a minimal parameter overhead.

(4) Efficient real-time deployment on an edge computing platform was successfully demonstrated. By coupling TensorRT optimization with FP16 half-precision quantization on the Jetson Orin Nano platform, near-lossless precision migration was achieved. The complete end-to-end system maintained robust real-time inference throughput, confirming its feasibility and engineering efficacy for real-world automated strawberry-thinning robotic operations.

## 2. Materials and Methods

### 2.1. Image Acquisition

**Figure 2:**
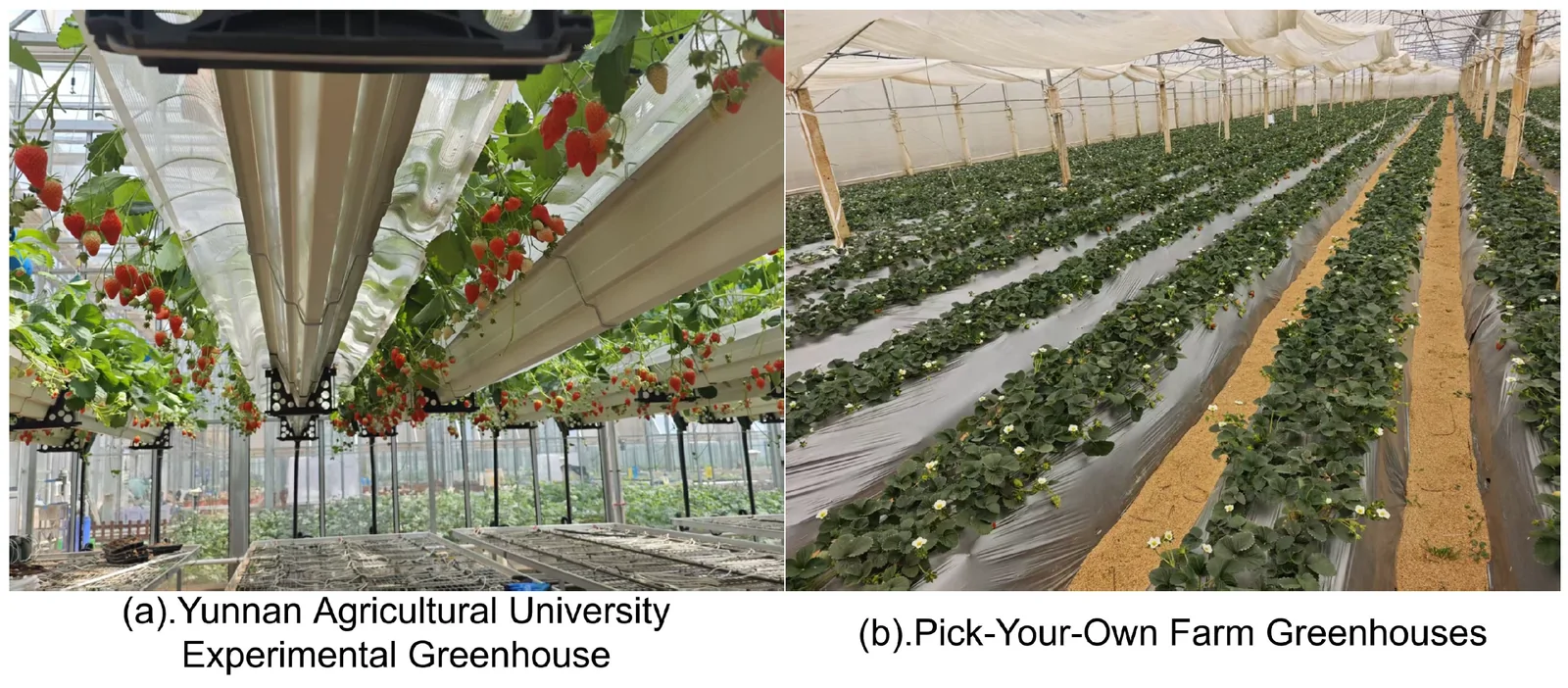
Comparison of differentiated strawberry image data collection scenarios.

To enhance the robustness and cross-scenario generalization capability of the instance segmentation model, image datasets were acquired from two distinct agricultural cultivation environments. The first dataset was collected at the experimental glass greenhouse facility of Yunnan Agricultural University, Kunming, Yunnan Province, China. In this facility, an elevated substrate cultivation system was adopted, characterized by relatively wide plant spacing and a comparatively structured spatial canopy arrangement. The two distinct strawberry acquisition environments are illustrated in Figure 2, with the elevated substrate system at Yunnan Agricultural University shown in Figure 2a and the pick-your-own farm greenhouse environment depicted in Figure 2b.

To encompass the complex environmental variations encountered in commercial production scenarios, additional images were collected from the Xiangye Strawberry Picking Orchard located in Qilin District, Qujing, Yunnan Province, China. In this environment, a conventional plastic-mulched soil ridge cultivation system was utilized. Compared with the elevated system, the strawberry plants were cultivated at a higher planting density, exhibiting substantial foliage interweaving, cluster overlapping, and mutual target occlusions. The detailed parameter settings and specifications for the image data acquisition are summarized in Table 1.

### 2.2. Dataset Annotation and Augmentation

**Figure 3:**
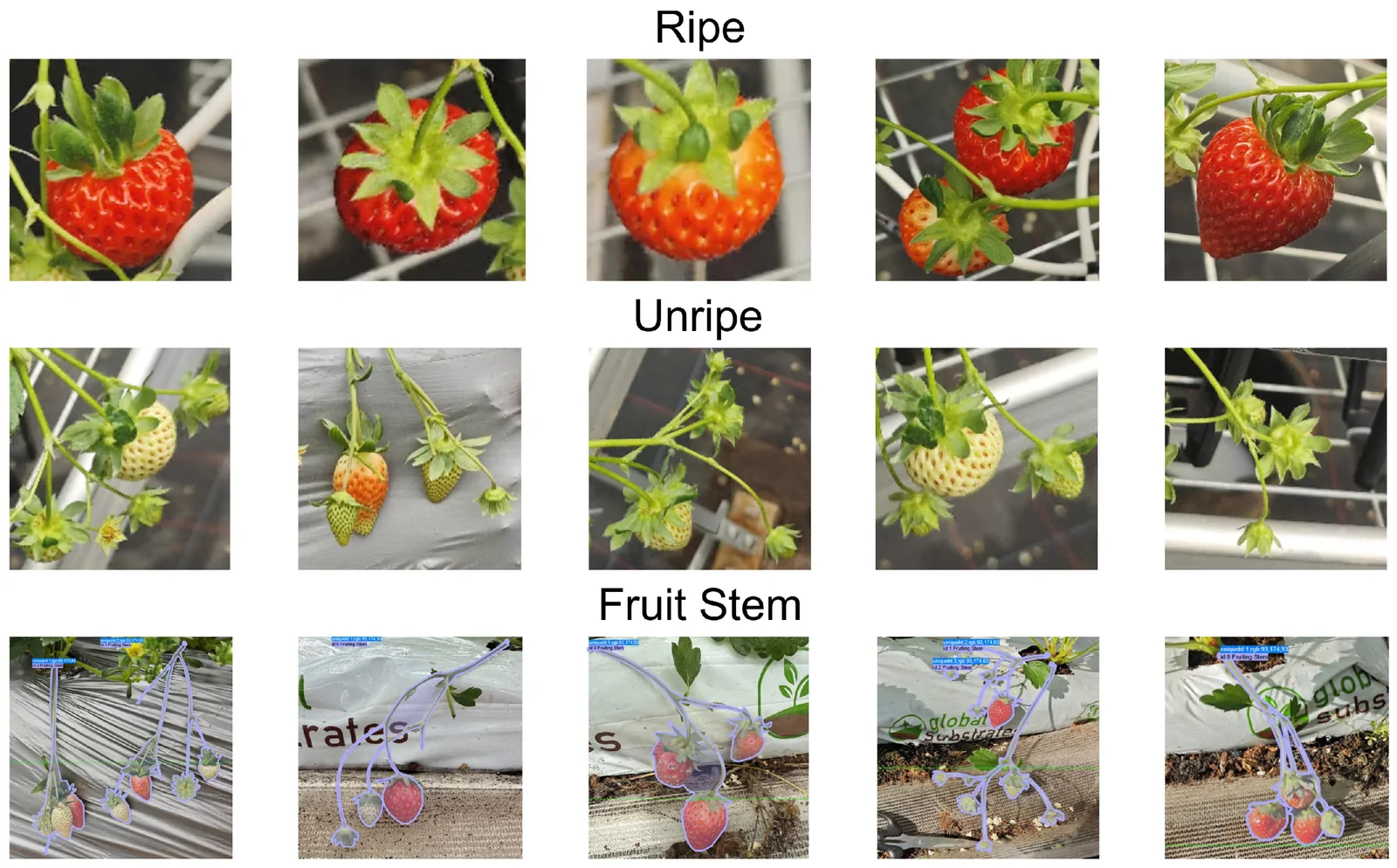
Sample examples of each category in the dataset.

All collected digital images were finely annotated using polygon-based instance segmentation labels. During the annotation process, polygon masks were manually delineated along the actual boundary contours of strawberry fruits and target peduncles, while background pixels within the annotated regions were minimized as much as possible. In this study, we focus on the topological association between strawberries and their peduncles. For simplicity and to maintain absolute consistency with the class names defined in our deep learning model and dataset annotations, the terms ‘fruit stem’ or simply ‘stem’ are used to refer to the peduncle in the following sections. Representative annotation examples for each defined category are illustrated in Figure 3.

The image acquisition campaign was conducted from December 2025 to March 2026. The acquired raw digital images were uniformly recorded at a high resolution of 4094×3072 pixels. To accurately replicate the operational visual conditions of automated fruit-thinning in field environments, the imaging sensor was horizontally positioned at a working distance of 30–50 cm from the center of the strawberry canopies. Multiple perspectives, including horizontal front-views, nadir top-views, and multi-directional side-views, were systematically acquired around the target plants. This acquisition strategy enriched the diversity of spatial geometric characteristics and illumination variations of strawberry organs, thereby establishing a robust benchmark for subsequent cascaded perception and fine-grained model training.

All acquired digital images were finely annotated with polygon-based instance segmentation masks. During the annotation process, boundary polygons were manually delineated along the precise morphological contours of strawberry fruits and target peduncles, minimizing background pixel contamination within the annotated masks. In this study, focus was placed on the topological association between strawberries and their supporting peduncles. For clarity and to maintain strict consistency with the class definitions in the deep learning model and dataset configuration, the term “fruit stem” (or simply “stem”) was adopted to denote the botanical peduncle in subsequent sections. Specifically, target instances were categorized into three distinct semantic classes: mature strawberries were annotated as “Ripe”, immature strawberries as “Unripe”, and target peduncle structures as “Fruit Stem”. The detailed category definitions and instance quantities for these annotation classes are summarized in Table 2.

**Figure 4:**
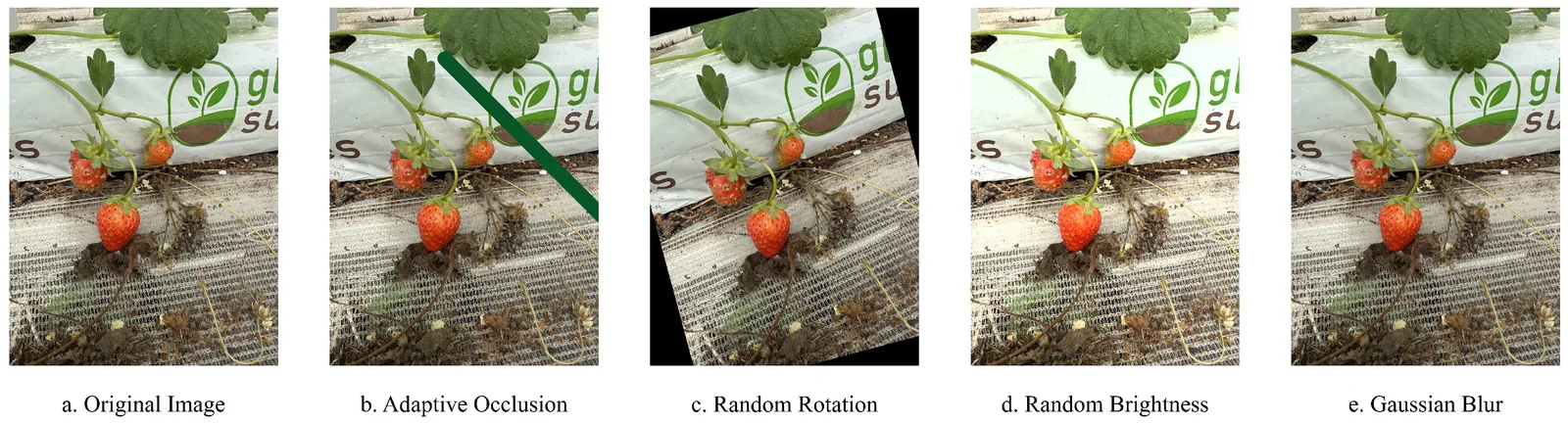
Schematic illustration of data augmentation effects.

To enhance the generalization capability and robustness of the automated fruit-thinning perception framework in complex, unstructured greenhouse environments, domain-specific data augmentation strategies were implemented on the annotated dataset. Specifically, an adaptive occlusion synthesis mechanism was applied to instance masks to simulate realistic occlusion patterns caused by randomly overlapping foliage and stems during field operations. This mechanism was designed to reinforce feature representation under severe occlusion and incomplete morphological cues. Random brightness adjustments were applied to simulate diurnal illumination fluctuations across diverse greenhouse structures. Random affine rotations were employed to simulate multi-angle viewpoint variations induced by the approaching trajectory of the robotic end-effector. Furthermore, random Gaussian blurring was incorporated to approximate optical defocusing and motion blur resulting from robotic arm manipulation and environmental disturbances, as illustrated in Figure 4.

Following data augmentation and strict dataset curation, a final benchmark instance segmentation dataset comprising 1131 images was constructed. The dataset was partitioned into a training set of 812 images (including augmented samples), a validation set of 159 images, and an independent testing set of 160 images, corresponding to an approximate ratio of 7:1.5:1.5. To prevent potential data leakage and ensure rigorous evaluation, the dataset was strictly split at the whole-image level rather than at the instance level. Furthermore, a stratified partitioning strategy was employed to ensure that an identical proportion of images from the two distinct greenhouse facilities was preserved across all three subsets. These subsets were subsequently deployed for model training, hyperparameter validation, and comparative performance evaluation. The detailed partitioning schemes, sample counts, and proportions of the dataset are presented in Table 3.

### 2.3. Proposed Method

**Figure 5:**
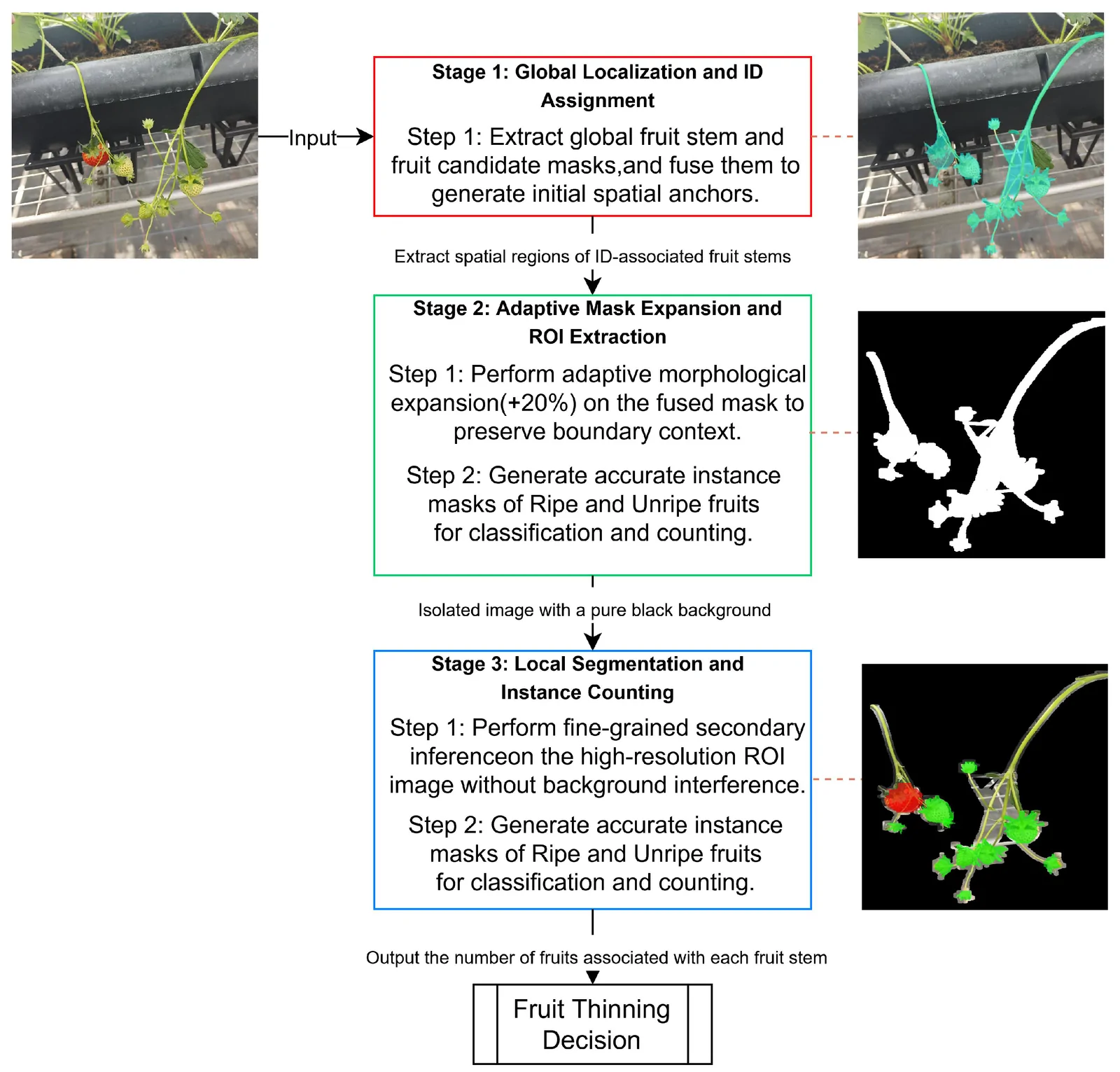
Flowchart of a hierarchical perception and segmentation process based on the coarse-to-fine strategy.

In dense greenhouse cultivation environments, severe occlusion caused by adjacent foliage and stems, as well as physical contact and mutual clustering between neighboring fruits, frequently induces feature confusion and erroneous instance assignment during single-stage forward inference. To mitigate complex background interference and enhance the feature representation of small-scale targets, a single-model, coarse-to-fine cascaded perception pipeline with localized attention was proposed in this study. The overall workflow is illustrated in Figure 5.

The complete original RGB image is initially processed as the input for the first-stage global coarse inference. The primary objective of this stage is not to extract boundary-precise segmentation contours, but rather to comprehensively detect all visible peduncles and candidate fruit instances across the global canopy. The preliminary segmentation masks generated at this stage serve as spatial anchors for subsequent local topological association. To prevent the boundary information of fruit clusters from being truncated during cropping, the peduncle and fruit masks predicted in Stage 1 are merged and adaptively expanded by 20%, utilizing a morphological dilation operation, thereby preserving adequate contextual semantics around the target boundaries.

Subsequently, complex background pixels outside the dilated mask regions are suppressed to zero intensity, and the minimum bounding rectangles enclosing the merged regions are extracted as regions of interest (ROIs) for sub-image cropping. The resulting localized high-resolution ROI images with zero-padded backgrounds are subsequently fed into the second inference stage. Because visual interference from surrounding non-target foliage and extraneous fruit clusters is effectively eliminated, the network’s feature attention is strictly focused on the spatial domain affiliated with the target peduncle. Consequently, refined instance segmentation masks for both mature (“Ripe”) and immature (“Unripe”) fruits are generated with substantially enhanced reliability.

Finally, the extracted fruit classification, peduncle–fruit topological correspondences, and quantitative load statistics are seamlessly transmitted to the downstream robotic automated fruit-thinning decision system.

#### 2.3.1. Global Localization and Mask Fusion

The primary objective of the first stage is to detect all potential targets from a global viewpoint and merge them into unified spatial regions. The original global image with a resolution of 4094×3072 pixels is initially fed into the lightweight instance segmentation network, RCA-YOLO, developed in this study (the detailed architecture is elaborated in Section 2.4). To minimize the risk of missed detections (false negatives), the confidence threshold of the network is configured to a relatively low value of 0.25. Three target categories—namely, peduncles (“Fruit Stem”), ripe fruits (“Ripe”), and unripe fruits (“Unripe”)—are detected simultaneously. At this stage, comprehensive target localization is prioritized over fine-grained boundary segmentation precision.

To establish unified spatial anchors, an empty mask canvas initialized with zero values is generated. During the traversal of detection outputs, a unique identifier (ID) is assigned to each detected peduncle instance. Subsequently, pixel-wise logical OR operations are performed to spatially merge all predicted fruit masks with their corresponding indexed peduncle masks, generating a fused base mask. The fusion process of the proposed mask is formulated as Equation (1).

(1) $$M_{\mathrm{base}}\left(x,y\right)=\overset{N}{\underset{i=1}{⋁}}S_{i}\left(x,y\right),\;\forall \left(x,y\right)\in \left[1,W\right]\times \left[1,H\right]$$

The generated fused base mask accurately demarcates the spatial territory of the entire strawberry cluster within the global field of view. To quantify the initial scale of this region and provide a geometric reference for the subsequent morphological expansion, the total number of valid foreground pixels enclosed by the fused mask is calculated, as defined in Equation (2).

(2) $$A_{\mathrm{orig}}=\sum_{x=1}^{W}\sum_{y=1}^{H}M_{\mathrm{base}}\left(x,y\right)$$

The mathematical symbols and notations involved in Equations (1) and (2) are explicitly defined in Table 4.

To explicitly formulate the proposed topological association mechanism between fruits and peduncles, the execution logic is summarized in Algorithm 1. Conventional, coarse bounding-box-based Intersection over Union (IoU) matching is superseded in this algorithm; instead, the minimum geometric Euclidean distances between polygon boundary point sets are evaluated to determine physical attribution. During execution, an adaptive dynamic distance threshold is dynamically computed based on the input image resolution and a predefined tolerance coefficient. Subsequently, all predicted fruit polygons are iteratively traversed, and the minimum Euclidean distances between their boundary contours and those of all candidate peduncle polygons are calculated to determine the nearest peduncle ID. Finally, strict gating is enforced via the dynamic distance threshold: if the shortest distance falls within the valid threshold boundary, the topological connection between the two entities is formally established; otherwise, the candidate fruit is categorized as detached background interference, and the association is explicitly rejected. This strategy achieves high-precision topological binding with minimal computational complexity, effectively filtering out environmental misclassifications at the logical level without necessitating complex post-processing networks.
**Algorithm 1** Topological association based on polygon distance**Require:** Image dimensions W×H, stem polygons $P_{\mathrm{stem}}$, fruit polygons $P_{\mathrm{fruit}}$, tolerance α **Ensure:** Fruit-to-stem assignment results A  1:  $T_{\mathrm{dist}}\leftarrow \max\left(W,H\right)\times \alpha$; A←∅ {Init threshold & output}  2:  **for each** $P_{\mathrm{fruit}}\in P_{\mathrm{fruit}}$ **do**  3:      $D_{i}\leftarrow \min\{{∥u-v∥}_{2}|u\in \partial P_{\mathrm{fruit}},v\in \partial P_{\mathrm{stem}}^{\left(i\right)}\}$ {Boundary distance}  4:      $ID_{\mathrm{best}}\leftarrow \arg\;\min_{i}\left(D_{i}\right)$; $\;D_{\min}\leftarrow \min_{i}\left(D_{i}\right)$  5:      **if** $D_{\min}\leq T_{\mathrm{dist}}$ **then**  6:          $A\left[P_{\mathrm{fruit}}\right]\leftarrow ID_{\mathrm{best}}$ {Valid topological connection}  7:      **else**  8:          $A[P_{\mathrm{fruit}}]\leftarrow -1$ {Blocked environmental interference}  9:      **end if**10:  **end for**11:  **return** A

The specific mathematical formulation of this association process is detailed as follows. First, to eliminate scale discrepancies caused by fixed pixel thresholds across devices with varying imaging resolutions, an adaptive dynamic distance threshold, $T_{\mathrm{dist}}$, is derived based on the spatial dimensions of the input image:

(3) $$T_{\mathrm{dist}}=\max\left(W,H\right)\times \alpha$$

where *W* and *H* represent the width and height of the input image in pixels, respectively, and α denotes the predefined topological connection tolerance coefficient (empirically set to 0.15 in this study). Subsequently, for any valid candidate fruit polygon, $P_{\mathrm{fruit}}$, predicted by the network, the set of all candidate peduncle polygons extracted from the global field of view, $P_{\mathrm{stem}}=\left\{P_{\mathrm{stem}}^{\left(1\right)},P_{\mathrm{stem}}^{\left(2\right)},\ldots ,P_{\mathrm{stem}}^{\left(n\right)}\right\}$, is traversed. The spatial distance, $D_{i}$, between the candidate fruit and the *i*-th candidate peduncle is defined as the minimum Euclidean distance between their boundary contours:

(4) $$D_{i}=\min\{{∥u-v∥}_{2}|u\in \partial P_{\mathrm{fruit}},v\in \partial P_{\mathrm{stem}}^{\left(i\right)}\}$$

Based on this metric, a greedy search strategy is employed to identify the optimal identifier of the nearest peduncle, ${\mathrm{ID}}_{\mathrm{best}}$, along with its corresponding minimum distance, $D_{\min}$:

(5) $${\mathrm{ID}}_{\mathrm{best}}=\underset{i}{\arg\;\min}\left(D_{i}\right)$$

(6) $$D_{\min}=\min_{i}\left(D_{i}\right)$$

Finally, the dynamic distance threshold is utilized to strictly gate this candidate connection from a physical topological perspective, yielding the definitive fruit attribution assignment, $A(P_{\mathrm{fruit}})$:

(7) $$A\left(P_{\mathrm{fruit}}\right)=\left\{\begin{array}{cc} {\mathrm{ID}}_{\mathrm{best}}, & \mathrm{if}\;D_{\min}\leq T_{\mathrm{dist}}\\ -1, & \mathrm{otherwise} \end{array}\right.$$

If the minimum distance, $D_{\min}$, is less than or equal to the threshold, $T_{\mathrm{dist}}$, the fruit instance is successfully assigned to the target peduncle, ${\mathrm{ID}}_{\mathrm{best}}$, establishing a verified topological connection. Conversely, if the distance exceeds the threshold (yielding an assignment output of −1), the two entities are deemed physically detached in space; the fruit is therefore classified as extraneous background foliage or neighboring plant interference, and the topological binding is explicitly rejected.

**Figure 6:**
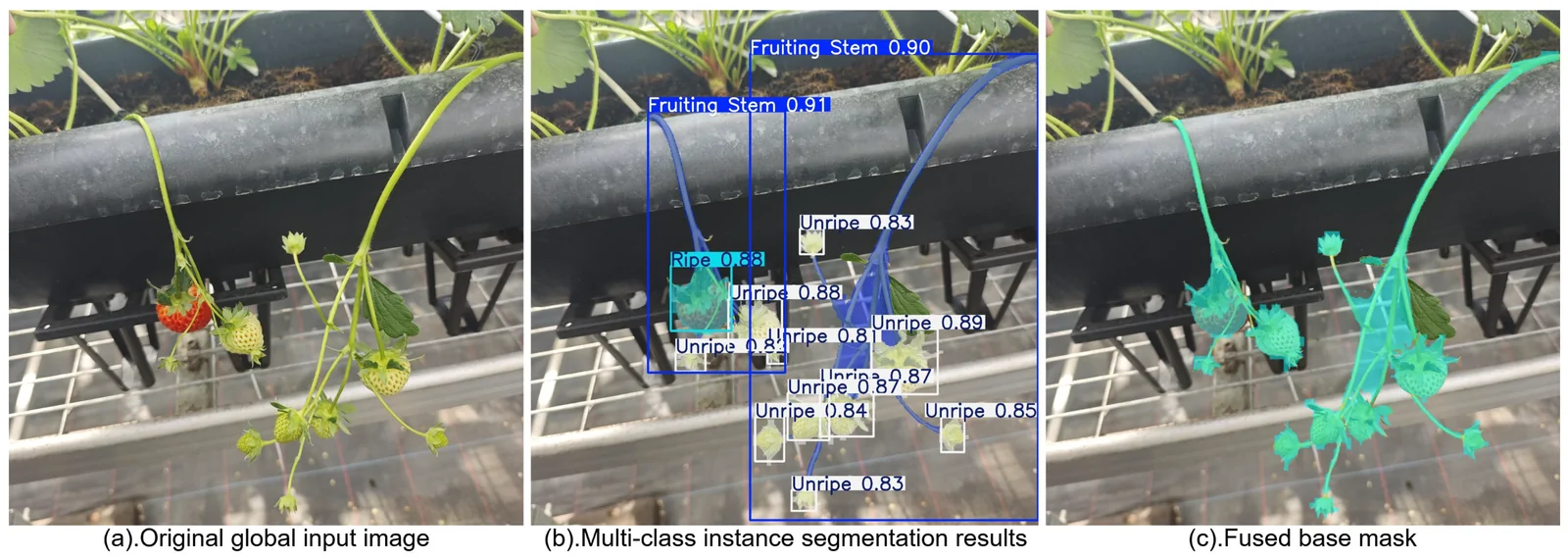
Global perception and mask fusion process in Stage 1.

The resulting fused base mask provides a reliable spatial constraint for the subsequent mask expansion and localized region cropping procedures. The overall global perception and mask fusion pipeline is schematically illustrated in Figure 6, showcasing the original input image (Figure 6a), the multi-class instance segmentation predictions (Figure 6b), and the resulting fused base mask (Figure 6c).

A critical operational challenge encountered during the initial stage is the potential false negative (FN) detection of slender peduncles. To address this issue, a conservative fail-safe strategy is integrated into the proposed perception pipeline. If a peduncle remains undetected during the global inference phase, the resultant incomplete fused base mask may induce erroneous region of interest (ROI) cropping and subsequent decision-making errors in the second stage. Consequently, processing for that specific fruit–peduncle cluster is deliberately terminated within the current frame, and the unverified target is discarded. This operational design strictly complies with the fundamental safety paradigm of agricultural robotics: the suppression of false positives is heavily prioritized over the minimization of false negatives. By proactively rejecting uncertain detections, irreversible and destructive robotic cutting actions (e.g., mistakenly severing the main vegetative stem) are effectively mitigated. Furthermore, facilitated by the continuous, multi-view dynamic perception capability of the moving robotic arm, these temporarily omitted targets are consistently recaptured in subsequent frames from alternative perspectives.

#### 2.3.2. Background Suppression and Local ROI Extraction

The primary objective of the second stage is to eliminate environmental background interference surrounding the target clusters and transform the global field of view into a localized high-resolution representation. The specific procedures for mask expansion and localized region cropping are summarized in Algorithm 2.
**Algorithm 2** Adaptive mask expansion and ROI extraction (Stage 2)**Require:** Global raw image $I_{\mathrm{global}}$, Fused global base mask $M_{\mathrm{base}}$ **Ensure:** Local high-resolution cropped ROI image $I_{\mathrm{ROI}}$  1:  Initialize the expanded mask canvas: $M_{\mathrm{expanded}}\leftarrow M_{\mathrm{base}}$  2:  {1. Adaptive morphological expansion}  3:  **repeat**  4:      Morphologically dilate $M_{\mathrm{expanded}}$ using a 5×5 ellipse kernel  5:  **until** Area of $M_{\mathrm{expanded}}$ increases by 20%  6:  {2. Background suppression}  7:  $I_{\mathrm{suppressed}}\leftarrow \mathrm{BitwiseAND}\left(I_{\mathrm{global}},\mathrm{mask}=M_{\mathrm{expanded}}\right)$  8:  {3. Region of Interest (ROI) cropping}  9:  Compute the minimum bounding rectangle of $M_{\mathrm{expanded}}$ with 2% safety padding10:  Crop the focused local image from $I_{\mathrm{suppressed}}$ to generate $I_{\mathrm{ROI}}$11:  **return** $I_{\mathrm{ROI}}$

First, a 5×5 elliptical structuring element is iteratively applied to dilate the fused base mask generated in Stage 1 until the foreground mask area reaches 120% of its initial scale (i.e., a 20% morphological expansion). This operation is strategically designed to preserve crucial contextual boundaries around the strawberry clusters, thereby preventing the truncation of peripheral edge features during subsequent processing.

Subsequently, a pixel-wise bitwise AND masking operation is executed between the expanded binary mask and the original RGB image. Consequently, all extraneous background pixels situated outside the dilated region of interest are suppressed to zero intensity (zero-padded). Through this localized filtering, visual interference induced by adjacent non-target foliage, overlapping stems, and complex greenhouse backgrounds is effectively isolated.

**Figure 7:**
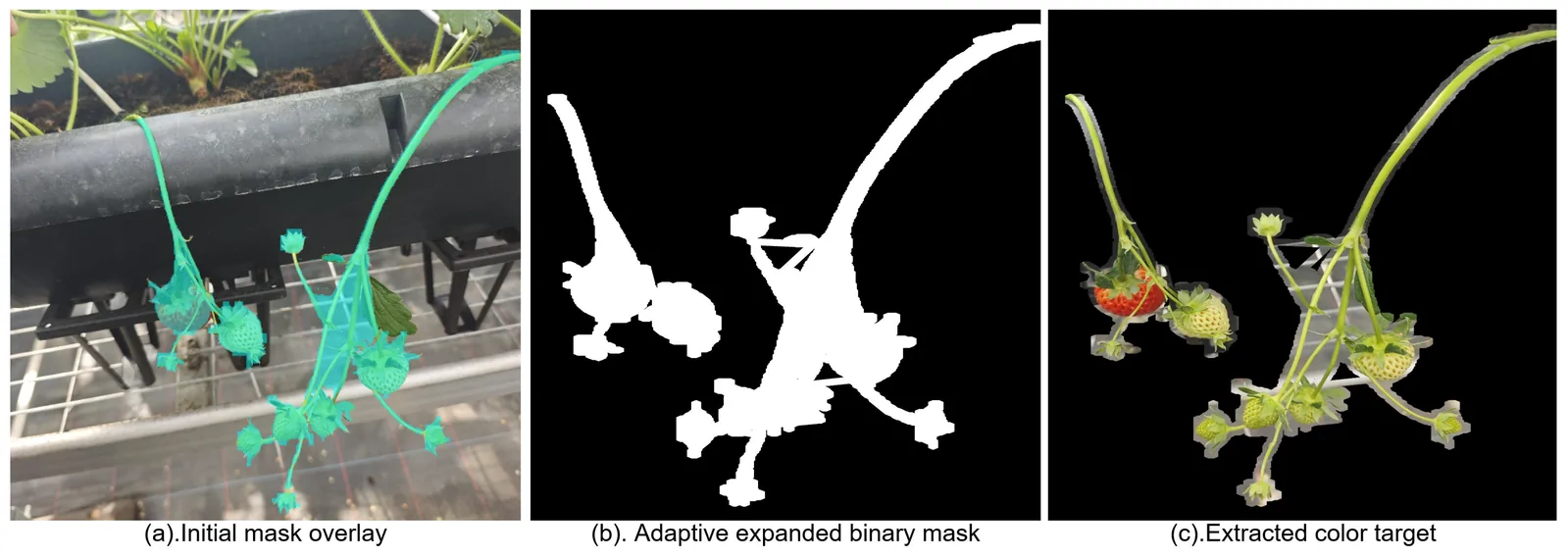
Background suppression and ROI extraction process.

Finally, the minimum bounding rectangle enclosing the dilated foreground region is computed, and an additional 2% outward spatial margin is incorporated as a cropping buffer. The resulting localized high-resolution ROI sub-image, containing exclusively the target strawberry cluster, is directly forwarded as the input for fine-grained secondary inference in Stage 3.The progressive pipeline of background suppression and ROI extraction is demonstrated in Figure 7, depicting the initial mask overlay (Figure 7a), the adaptive expanded binary mask (Figure 7b), and the final extracted color target with the background removed (Figure 7c). The background suppression and ROI extraction workflow is illustrated in Figure 7.

As presented in Table 5, a parameter sensitivity analysis was performed on the validation set to validate the empirical selection of hyperparameters. For the morphological mask expansion, a 20% expansion ratio proved optimal: lower expansion levels (e.g., 10%) tend to truncate peripheral features and reduce recall, whereas excessive ratios (e.g., 30%) incorporate excessive background clutter that degrades the overall mAP. Similarly, a 2% boundary buffer achieved the highest mAP@0.5. A 0% buffer leads to the degradation of boundary features during ROI extraction, whereas buffer margins exceeding 2% introduce extraneous neighboring structures that compromise segmentation precision.

#### 2.3.3. Local Fine-Grained Segmentation and Individual Fruit Counting

The primary objective of the third stage is to achieve precise instance segmentation and quantitative enumeration of strawberries within the background-suppressed localized images. The region of interest (ROI) sub-images featuring zero-padded backgrounds are re-fed into the identical neural network architecture, which inherently possesses the capability for joint recognition across three semantic categories (peduncles, ripe fruits, and unripe fruits). To reduce classification ambiguity and optimize inference efficiency, a target class filtering parameter (i.e., specifying classes=[1, 2]) is directly configured at the API invocation level of the YOLO framework. Consequently, candidate peduncle proposals are suppressed at the source during the underlying Non-Maximum Suppression (NMS) procedure. Through this engineering optimization, the inference space is strictly restricted to two categories—mature fruits (“Ripe”) and immature fruits (“Unripe”)—thereby obviating the requirement for redundant post-processing filtering operations. The detailed execution pipeline of this stage is delineated in Algorithm 3.
**Algorithm 3** Local fine instance segmentation (Stage 3)**Require:** Suppressed ROI image $I_{\mathrm{roi}}$, Segmentation Model *F* **Ensure:** Fruit counts ($N_{\mathrm{ripe}},N_{\mathrm{unripe}}$), Visualized output $I_{\mathrm{final}}$  1:  Initialize $N_{\mathrm{ripe}}\leftarrow 0$, $N_{\mathrm{unripe}}\leftarrow 0$, $I_{\mathrm{final}}\leftarrow I_{\mathrm{roi}}$  2:  $P_{\mathrm{local}}\leftarrow F\left(I_{\mathrm{roi}},\mathrm{classes}=\left\{\mathrm{Ripe},\mathrm{Unripe}\right\}\right)$  3:  **for** each instance in $P_{\mathrm{local}}$ **do**  4:      $M_{\mathrm{bin}}\leftarrow \mathrm{Binarize}\left(\mathrm{Resize}\left(\mathrm{instance}.\mathrm{mask},\mathrm{size}\left(I_{\mathrm{roi}}\right)\right)\right)$  5:      **if** instance.class==RIPE **then**  6:          $N_{\mathrm{ripe}}\leftarrow N_{\mathrm{ripe}}+1$  7:      **else**  8:          $N_{\mathrm{unripe}}\leftarrow N_{\mathrm{unripe}}+1$  9:      **end if**10:       $I_{\mathrm{final}}\leftarrow \mathrm{Overlay}\text{\_}\mathrm{Mask}\text{\_}\mathrm{and}\text{\_}\mathrm{Contours}\left(I_{\mathrm{final}},M_{\mathrm{bin}}\right)$11:  **end for**12:  **return** $N_{\mathrm{ripe}},N_{\mathrm{unripe}},I_{\mathrm{final}}$

Benefiting from the effective suppression of non-target environmental clutter, such as adjacent foliage and extraneous stems, the network’s feature attention is concentrated on localized fruit clusters with minimal visual interference. Following inference, the detected instances are automatically traversed for semantic classification and fruit-load enumeration. Concurrently, precise instance boundaries are extracted through mask resizing and threshold binarization.

**Figure 8:**
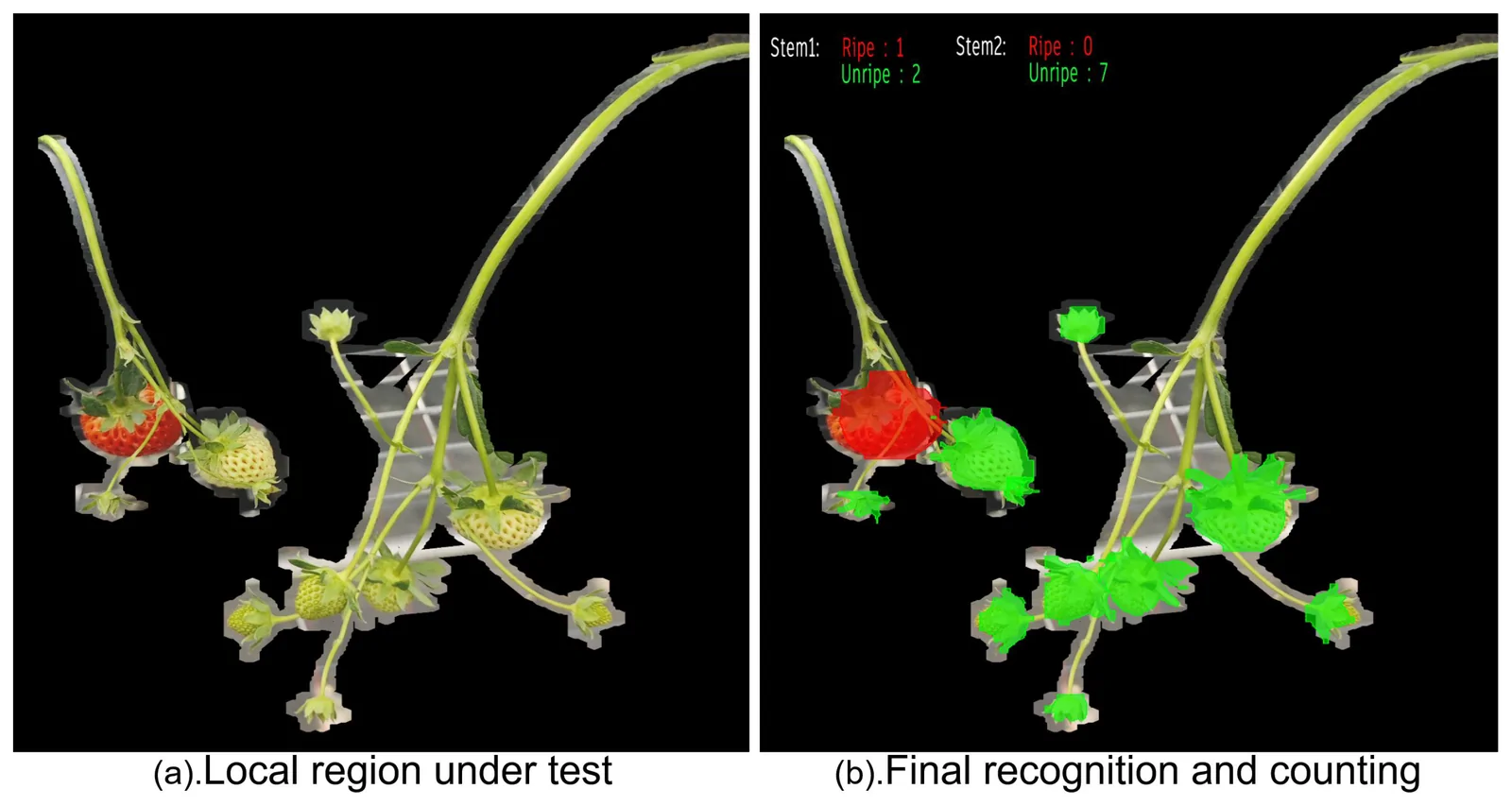
Precision segmentation and counting comparison in Stage 3.

A qualitative comparison of the visual results before and after secondary inference is illustrated in Figure 8. Figure 8a depicts the cropped ROI containing exclusively the target cluster against a suppressed background, whereas Figure 8b displays the final segmentation masks and topological enumeration outcomes generated by the secondary inference stage. The proposed framework not only generates fine-grained fruit masks that conform closely to actual morphological contours, but also provides real-time hierarchical counting metrics.

These accurate visual segmentations and quantitative statistics are directly integrated into the downstream intelligent robotic decision-making system, establishing a reliable operational baseline for automated strawberry fruit-thinning operations.

### 2.4. RCA-YOLO Segmentation Model

**Figure 9:**
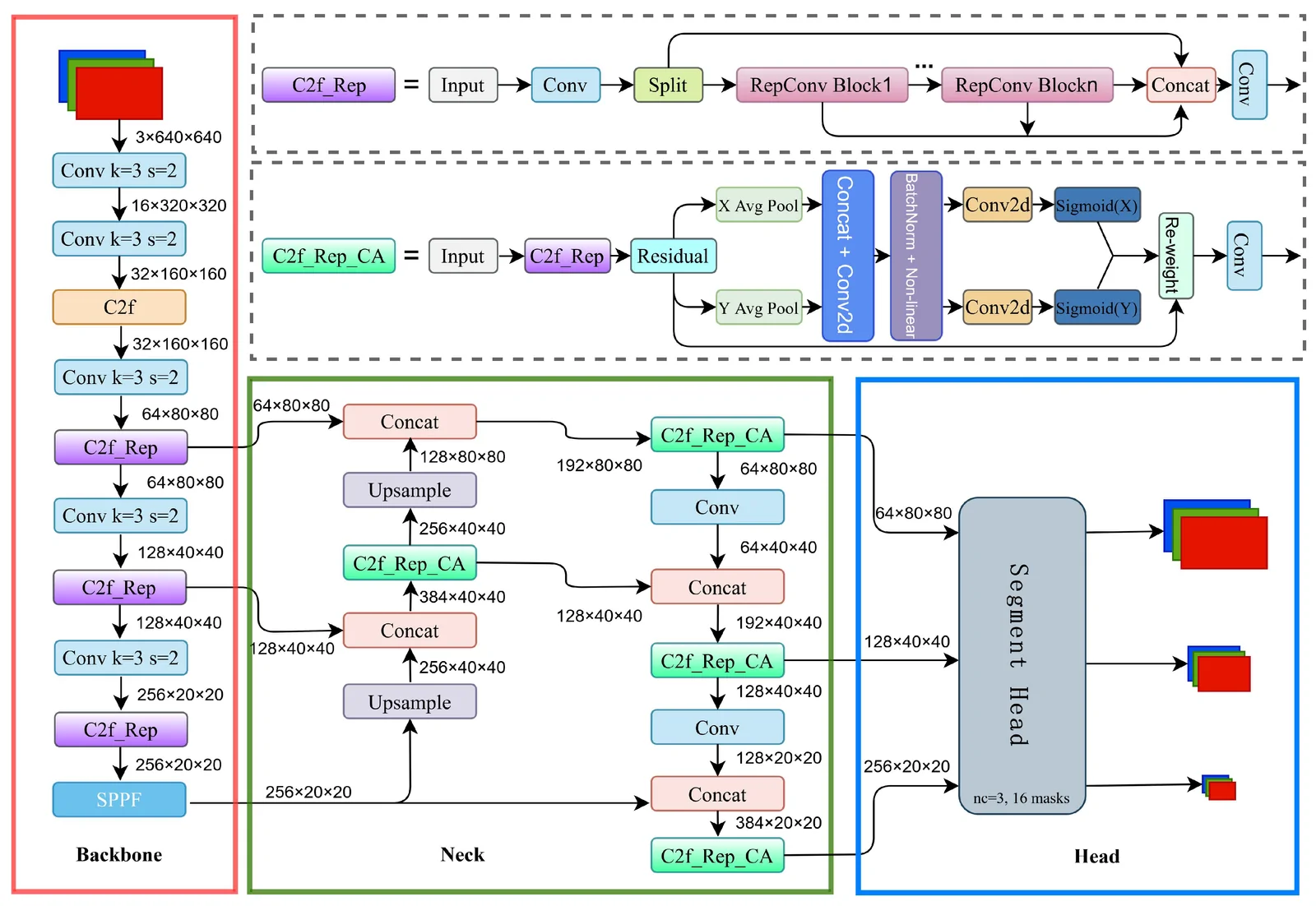
The overall network architecture of the proposed RCA-YOLO model, with detailed structures of C2f_Rep and C2f_Rep_CA shown in dashed boxes.

To achieve accurate and real-time instance segmentation on resource-constrained edge computing devices (e.g., NVIDIA Jetson), a lightweight, spatially coordinate-aware network, designated as RCA-YOLO (RepConv and Coordinate Attention YOLO), was developed based on the YOLO11n-seg baseline. The overall network architecture is schematically illustrated in Figure 9.

In the backbone feature extraction network, a streamlined C2f_Rep module was engineered by incorporating Structural Re-parameterized Convolution (RepConv). By exploiting the structural re-parameterization paradigm—where multi-branch topologies are utilized during training and equivalent single-branch convolutions are merged during inference—the proposed module accelerates inference throughput while preserving rich feature gradient propagation. Concurrently, the computationally intensive C2PSA module was eliminated to further reduce peak memory overhead and latency.

To address the challenges posed by slender, highly occluded peduncles in dense strawberry canopies, a novel C2f_Rep_CA fusion module was incorporated into the neck feature aggregation network. This module deeply integrates the Coordinate Attention (CoordAtt) mechanism, wherein spatial orientation information is aggregated via 1D positional pooling along both the horizontal and vertical directions. Consequently, the network’s capability to capture directional topological features and long-range spatial dependencies of peduncles is significantly strengthened, thereby mitigating false negatives in complex overlapping cluster regions.

For mask generation and scaling configuration, RCA-YOLO adopts a decoupled Segment Head to process multi-scale aggregated feature pyramids ($P_{3}$–$P_{5}$). Bounding-box predictions, semantic class probabilities, and 16 prototypical mask coefficients are concurrently generated via parallel decoupled branches. To achieve an optimal trade-off between segmentation precision and edge deployment efficiency, the entire architecture adopts a Nano-scale scaling configuration with a width multiplier of 0.25 and a depth multiplier of 0.33. This lightweight configuration substantially minimizes the parameter count and floating-point operations (FLOPs), enabling low-latency, real-time operation of the automated fruit-thinning robotic system on physical edge platforms.

#### 2.4.1. Structural Re-Parameterized Convolution Module (RepConv)

**Figure 10:**
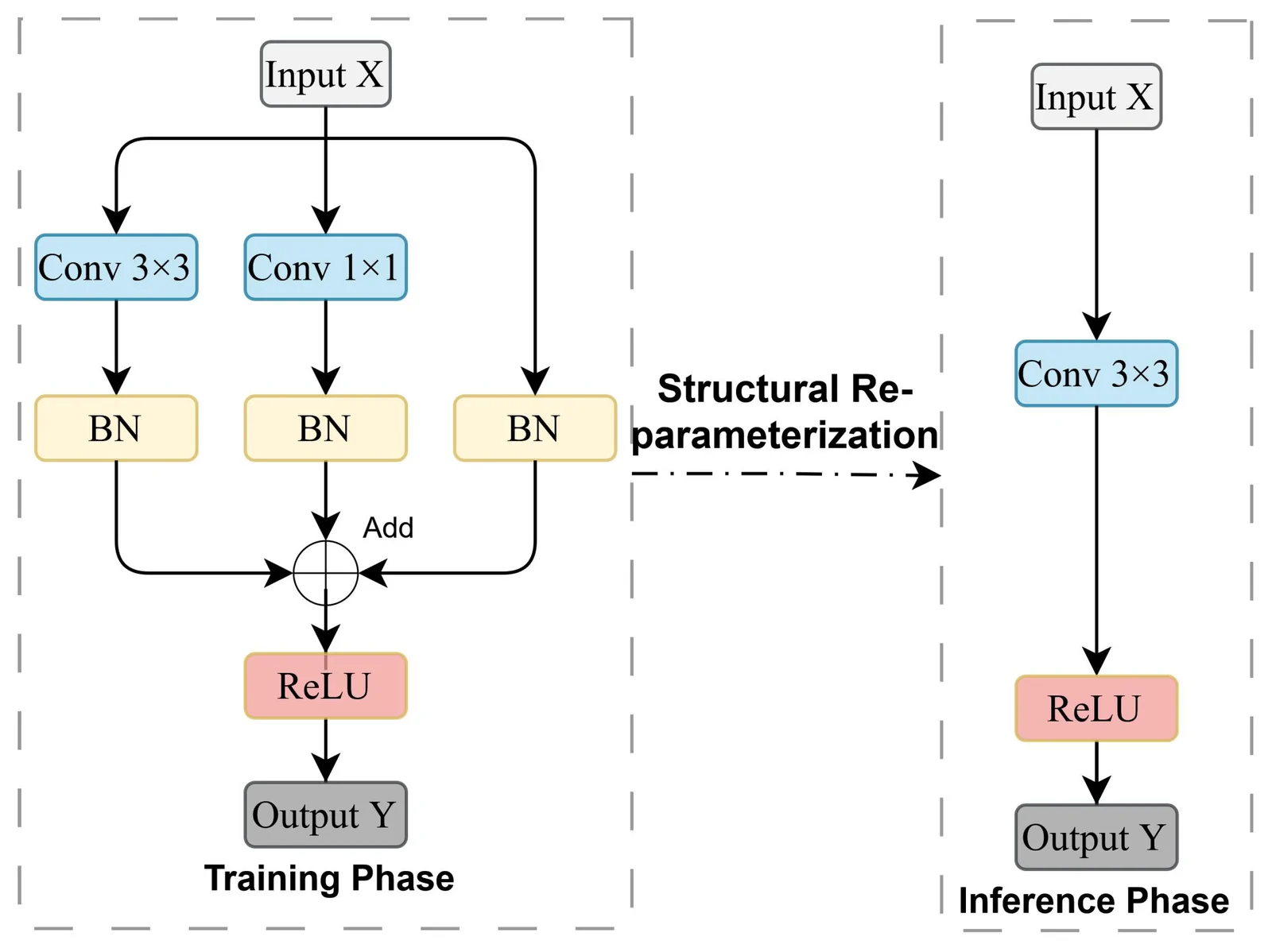
Schematic diagram of the structural re-parameterization process in the RepConv module.

Although conventional multi-branch network architectures provide robust feature representation capabilities, their fragmented topological structures introduce an increased memory access cost (MAC) during inference, resulting in latency penalties on edge computing devices. To alleviate this limitation, a Structural Re-parameterized Convolution (RepConv) module was incorporated into the feature extraction network. The underlying feature representation process was restructured via a decoupled training-inference paradigm, utilizing a multi-branch topology during training and a single-path structure during inference, as illustrated in Figure 10.

During the training phase, RepConv adopts a multi-branch configuration to enhance multi-scale feature representation and gradient backpropagation. Specifically, given an input feature tensor *X*, the RepConv module concurrently computes outputs across three parallel branches: a 3×3 standard convolution branch, a 1×1 point-wise convolution branch, and an identity residual mapping branch (activated exclusively when the input and output channel dimensions and spatial resolutions are identical). Each branch is immediately followed by a Batch Normalization (BN) layer. The resulting output of RepConv during the training phase, denoted as $Y_{\mathrm{train}}$, is mathematically formulated as Equation (8).

(8) $$Y_{\mathrm{train}}=\mathrm{BN}\left({\mathrm{Conv}}_{3\times 3}\left(X\right)\right)+\mathrm{BN}\left({\mathrm{Conv}}_{1\times 1}\left(X\right)\right)+\mathrm{BN}\left(X\right)$$

This parallel structural configuration can be interpreted as a linear combination of feature representations across distinct receptive fields, facilitating gradient optimization in deep layers and augmenting the model’s capacity to extract fine-grained morphological features of strawberry fruits and peduncles.

During edge deployment and inference, both convolution operations and BN layers are mathematically characterized as linear transformations. Consequently, RepConv leverages operator fusion to equivalently re-parameterize the multi-branch training structure into a single, highly efficient 3×3 convolution layer.

First, the parameters of the BN layers across each branch—including the scaling factor γ, bias term β, running mean μ, and variance $\sigma^{2}$—are fused into the corresponding convolution kernel weights *W* and bias terms *b*. The equivalent fused convolution weights $W^{\prime }$ and bias terms $b^{\prime }$ are calculated in accordance with Equations (9) and (10).

(9) $$W^{\prime }=\frac{\gamma }{\sqrt{\sigma^{2}+\epsilon }}W$$

(10) $$b^{\prime }=\beta -\frac{\gamma \mu }{\sqrt{\sigma^{2}+\epsilon }}$$

Subsequently, by utilizing the linear additivity of convolution operators, the 1×1 convolution kernel is geometrically expanded into a 3×3 kernel via spatial zero-padding. Concurrently, the identity mapping is transformed into an equivalent 3×3 convolution kernel, wherein the central element is assigned a unit weight of 1 and all peripheral elements are set to 0. Finally, the three transformed convolution kernels and their corresponding bias vectors are fused via element-wise addition, yielding a unified, equivalent 3×3 convolution kernel deployed for real-time inference.

#### 2.4.2. Coordinate Attention Mechanism (CoordAtt)

In the proposed decision-making system, peduncles and overlapping fruit clusters are treated as critical visual targets. However, peduncles typically exhibit slender morphological structures and are prone to severe occlusion by dense foliage and adjacent fruits under unstructured greenhouse conditions. Conventional channel attention mechanisms generally rely on 2D global average pooling to compress spatial feature maps into 1D channel descriptors. Although such operations effectively capture inter-channel dependencies, spatial positional information is inevitably discarded, making it difficult to preserve the precise topological features of slender targets.

**Figure 11:**
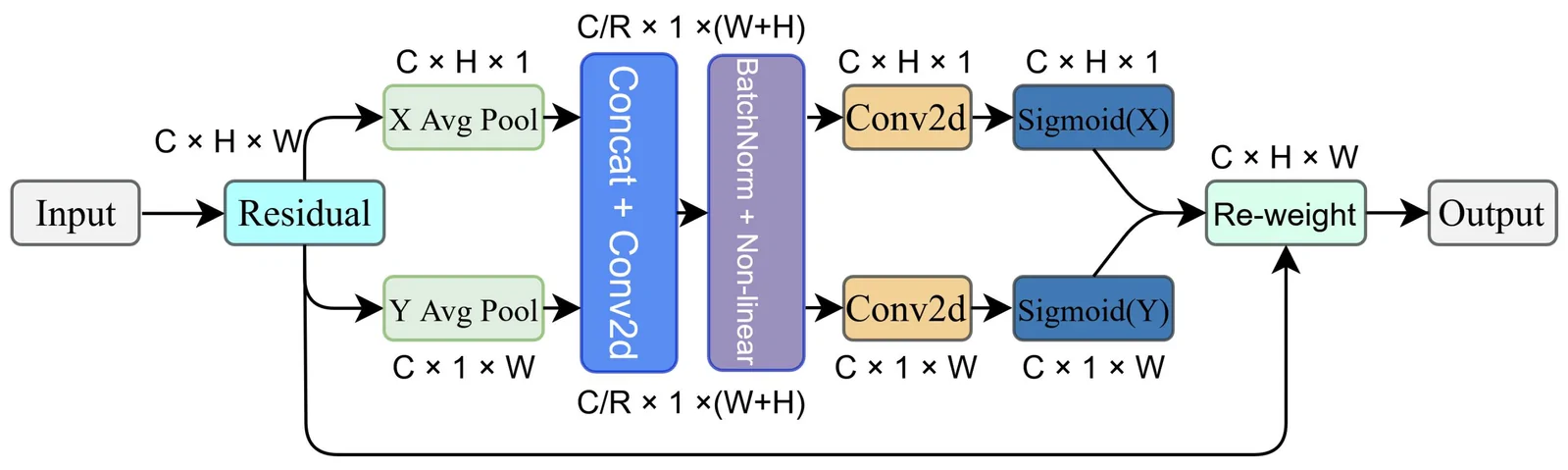
Detailed structure of the RCA module.

To overcome this limitation, a Coordinate Attention (CoordAtt) mechanism was incorporated into the neck feature fusion network. The detailed structural pipeline of the attention module is illustrated in Figure 11. The core principle of CoordAtt is to factorize conventional 2D global pooling into two parallel 1D feature encoding operations along the horizontal (*X*-axis) and vertical (*Y*-axis) directions. This directional decoupling strategy not only models cross-channel relationships, but also preserves precise spatial coordinate information across long-range dependencies.

The computational workflow of CoordAtt comprises three primary phases. First, direction-aware pooling is executed on the input feature tensor with spatial dimensions of C×H×W utilizing pooling kernels of sizes 1×W and H×1 along the horizontal and vertical directions, respectively. For an input feature tensor *X*, the encoded output of the *c*-th channel at height coordinate *h* and width coordinate *w* is formulated as shown in Equations (11) and (12), respectively.

(11) $$z_{c}^{h}\left(h\right)=\frac{1}{W}\sum_{0\leq i<W}x_{c}\left(h,i\right)$$

(12) $$z_{c}^{w}\left(w\right)=\frac{1}{H}\sum_{0\leq j<H}x_{c}\left(j,w\right)$$

Subsequently, the two generated directional feature maps are concatenated along the spatial dimension and fed into a shared 1×1 convolution layer for channel reduction with a compression ratio of *r*. Following Batch Normalization and a non-linear activation function, an intermediate spatial-channel feature tensor is yielded: $f\in R^{C/r\times 1\times (W+H)}$.

(13) $$f=\delta \left({\mathrm{Conv}}_{1\times 1}\left([z^{h},z^{w}]\right)\right)$$

The intermediate tensor f is subsequently partitioned along the spatial dimension into two distinct directional tensors, $f^{h}\in R^{C/r\times H\times 1}$ and $f^{w}\in R^{C/r\times 1\times W}$. These tensors are separately passed through two independent 1×1 convolution layers to recover the original channel dimension *C*. Thereafter, the Sigmoid activation function is applied to yield the vertical and horizontal attention weight matrices, $g^{h}$ and $g^{w}$, respectively:

(14) $$g^{h}=\sigma \left({\mathrm{Conv}}_{h}\left(f^{h}\right)\right)$$

(15) $$g^{w}=\sigma \left({\mathrm{Conv}}_{w}\left(f^{w}\right)\right)$$

Finally, as illustrated in the residual branch of Figure 11, the original feature flow is adaptively re-weighted via element-wise multiplication with the two orthogonal directional attention matrices. Through the broadcasting mechanism, pixel-wise feature recalibration is achieved, producing the final recalibrated output, $y_{c}\left(i,j\right)$:

(16) $$y_{c}\left(i,j\right)=x_{c}\left(i,j\right)\times g_{c}^{h}\left(i\right)\times g_{c}^{w}\left(j\right)$$

#### 2.4.3. Introduction of Focal Tversky Loss to Mitigate Extreme Sample and Spatial Imbalance

During the construction of the strawberry dataset, an extreme class imbalance was observed: the number of peduncles is significantly lower than that of fruits, and peduncles possess an inherently slender morphological structure. In standard instance segmentation training, when conventional Binary Cross-Entropy (BCE) or Dice loss is employed, gradient backpropagation is easily dominated by the abundant fruit pixels and background regions. This dual imbalance in both sample quantity and spatial pixel scale frequently leads to boundary fragmentation or severe false negatives (missed detections) when segmenting slender peduncles.

To address this challenge, the mask segmentation loss function of RCA-YOLO was restructured by incorporating the Focal Tversky Loss (FTL) mechanism. The Tversky index overcomes the limitation of traditional Intersection over Union (IoU) metrics, which treat false positives (FP) and false negatives (FN) equally, by independently adjusting their penalty weights. Building on this, FTL introduces a focusing mechanism that compels the network to focus on hard-to-segment peduncle boundary pixels. The mathematical formulations are defined in Equations (17) and (18):

(17) $$T\left(p,g\right)=\frac{\mathrm{TP}}{\mathrm{TP}+\alpha \mathrm{FN}+\beta \mathrm{FP}+\epsilon }$$

(18) $$L_{FT}={\left(1-T(p,g)\right)}^{\gamma }$$

where TP, FN, and FP denote pixel-level true positives, false negatives, and false positives, respectively, and ϵ represents a smoothing factor to prevent division by zero. Regarding hyperparameter configurations, α and β were set to 0.75 and 0.25, respectively. By assigning a higher value to α, a larger penalty was imposed on the missed detection (FN) of peduncles. Simultaneously, the focusing parameter γ was set to 2.0 to adaptively suppress gradient contributions from easy-to-segment pixels.

Notably, to further enhance the detection of minority classes, a class-aware dynamic re-weighting strategy was designed for the loss computation phase. During the loss calculation for each image, ground-truth class labels are automatically extracted. When a target is identified as a peduncle (class index 0), the computed Focal Tversky loss is superimposed onto the baseline BCE loss with an enhanced penalty weight of 1.5. This combined strategy of pixel-level focusing and class-level re-weighting effectively mitigates the network’s tendency to overlook sparse, slender targets, thereby preventing the practical defect of only detecting fruits while missing supporting peduncles during robotic deployment.

## 3. Results

### 3.1. Experimental Environment

The hardware platform employed in this study was configured with an Intel Xeon Platinum 8368Q @ 2.60 GHz dual-socket server-grade processor and an NVIDIA GeForce RTX 5090 graphics processing unit (GPU) equipped with 32 GB of VRAM. The system memory (RAM) was configured to 128 GB. All experiments were conducted on a 64-bit Windows 10 Professional operating system. The software environment was established based on Python 3.11.15, with model development and training implemented using the PyTorch 2.12.0 deep learning framework and accelerated via CUDA 12.9.

Regarding the hyperparameter configurations for model training, the input image resolution was uniformly set to 640×640 pixels. The training batch size was set to 32, and the models were trained for a total of 300 epochs. To accommodate the integrated RepConv and CoordAtt mechanisms and prevent early gradient explosion, the AdamW optimizer was adopted, with an initial learning rate set to 0.001. A warm-up strategy was implemented during the first five epochs, after which the learning rate was decayed following a cosine annealing schedule with a final learning rate fraction (*lrf*) of 0.01. For the loss components, the loss gain weights for bounding box regression, classification, and distribution focal loss (DFL) were configured to 5.0, 2.5, and 1.5, respectively. Additionally, label smoothing with a coefficient of 0.2 was applied to alleviate overfitting in complex background scenarios. The detailed experimental hardware and software environment configurations are summarized in Table 6.

Regarding online data augmentation strategies, since domain-specific operations such as localized perspective simulation had already been completed during the offline stage, geometric transformations during online training were moderately constrained (the rotation angle was set to 0, and the scaling factor was limited to 0.1). Meanwhile, Copy-Paste augmentation with an execution probability of 0.6 and Mosaic augmentation were activated to further enrich the scale diversity of peduncles and fruits. To promote stable model convergence toward the true physical scales of the targets during the late training stage, Mosaic augmentation was disabled during the final 10 epochs. The specific hyperparameter configurations for network training are listed in Table 7.

### 3.2. Evaluation Metrics

To objectively and comprehensively evaluate the detection and segmentation performance of the improved RCA-YOLO model on strawberry fruits and slender peduncles in densely occluded scenarios, standard evaluation metrics in object detection and instance segmentation were selected: Precision, Recall, F1-score, and Mean Average Precision (mAP).

Precision measures the proportion of true positive targets among all samples predicted as the positive class, reflecting the accuracy of the model. Conversely, Recall measures the proportion of ground-truth targets successfully detected by the model, reflecting the detection completeness (resistance to missed detections). Their mathematical formulations are expressed as Equations (19) and (20), respectively:

(19) $$\mathrm{Precision}=\frac{\mathrm{TP}}{\mathrm{TP}+\mathrm{FP}}$$

(20) $$\mathrm{Recall}=\frac{\mathrm{TP}}{\mathrm{TP}+\mathrm{FN}}$$

where TP (true positive) denotes the number of correctly predicted strawberry fruits or peduncles; FP (false positive) represents background, foliage, or extraneous objects misclassified as positive targets (false detections); and FN (false negative) denotes the number of true targets omitted by the network (missed detections).

As previously stated, an extreme class imbalance exists in the quantitative distribution between fruits and peduncles under actual greenhouse conditions. To prevent evaluation bias caused by relying on a single metric under data skewness, the F1-score was introduced as a harmonic evaluation criterion. The F1-score represents the harmonic mean of precision and recall, balancing both metrics to reflect model robustness under sample imbalance. Its mathematical definition is presented in Equation (21):

(21) $$F1=2\times \frac{\mathrm{Precision}\times \mathrm{Recall}}{\mathrm{Precision}+\mathrm{Recall}}$$

Average precision (AP) is defined as the area under the precision–recall (P–R) curve, plotted with recall (*R*) on the horizontal axis and precision (*P*) on the vertical axis. It provides a comprehensive evaluation of detection performance for an individual category; mAP is the mean of the AP values across all semantic classes. In this study, mAP@0.5 is primarily adopted, representing the mean average precision calculated at an Intersection over Union (IoU) threshold of 0.50. The formulations are defined in Equations (22) and (23):

(22) $$\mathrm{AP}=\int_{0}^{1}P\left(R\right)\;dR$$

(23) $$\mathrm{mAP}=\frac{1}{N}\sum_{i=1}^{N}{\mathrm{AP}}_{i}$$

where P(R) denotes the precision function parameterized by recall *R*, and *N* represents the total number of semantic categories (N=3 in this study, corresponding to ripe fruits, unripe fruits, and peduncles). The mAP@0.5 metric intuitively reflects overall performance in multi-class organ localization and mask segmentation in complex backgrounds.

To further evaluate the system-level performance of the proposed multi-stage pipeline, topological evaluation metrics were established. The topological counting accuracy represents the system-level recall (${\mathrm{Recall}}_{\mathrm{topo}}$), measuring the proportion of true positive fruits (TP) successfully matched to their ground-truth supporting peduncles relative to the total number of annotated ground-truth fruits (GT). Its calculation formula is as follows:

(24) $${\mathrm{Acc}}_{\mathrm{topo}}={\mathrm{Recall}}_{\mathrm{topo}}=\left(\frac{\sum_{i=1}^{N_{\mathrm{img}}}{\mathrm{TP}}_{i}}{\sum_{i=1}^{N_{\mathrm{img}}}{\mathrm{GT}}_{i}}\right)\times 100\%$$

where $N_{\mathrm{img}}$ denotes the total number of test images.

Secondly, the topological precision (${\mathrm{Precision}}_{\mathrm{topo}}$) reflects the proportion of fruits with correctly established topological connections among all fruit predictions generated by the system (including true positives TP and false positive misassignments FP). The formula is expressed as follows:

(25) $${\mathrm{Precision}}_{\mathrm{topo}}=\left(\frac{\sum_{i=1}^{N_{\mathrm{img}}}{\mathrm{TP}}_{i}}{\sum_{i=1}^{N_{\mathrm{img}}}\left({\mathrm{TP}}_{i}+{\mathrm{FP}}_{i}\right)}\right)\times 100\%$$

Furthermore, to evaluate the balanced trade-off between false alarm suppression and true target retention, the topological F1-score ($F1_{\mathrm{topo}}$) was calculated as the harmonic mean of ${\mathrm{Precision}}_{\mathrm{topo}}$ and ${\mathrm{Recall}}_{\mathrm{topo}}$:

(26) $${\mathrm{mAP}}_{\mathrm{mask}}@0.5=\frac{2\times \mathrm{Precision}\text{\_}\mathrm{box}@0.5\times {\mathrm{Rec}}_{\mathrm{topo}}}{\mathrm{Precision}\text{\_}\mathrm{box}@0.5+{\mathrm{Rec}}_{\mathrm{topo}}}$$

Finally, to quantify the effectiveness of spatial filtering and background suppression in eliminating environmental clutter, the Average False Positives per Image (${\mathrm{FP}}_{\mathrm{avg}}$) was introduced. The false positives for a single image, ${\mathrm{FP}}_{i}$, include topological mismatches resulting from erroneous fruit-to-peduncle assignments and unsuppressed background artifacts. The average metric across *M* evaluation images is formulated as follows:

(27) $$\mathrm{Average}\;\mathrm{FP}=\frac{\sum_{i=1}^{N_{\mathrm{img}}}{\mathrm{FP}}_{i}}{N_{\mathrm{img}}}$$

A lower value of this metric indicates superior performance of the pipeline in suppressing complex background clutter and isolating valid target regions of interest.

### 3.3. Experimental Results and Analysis

#### 3.3.1. Performance Analysis of the Hierarchical Segmentation Strategy

**Figure 12:**
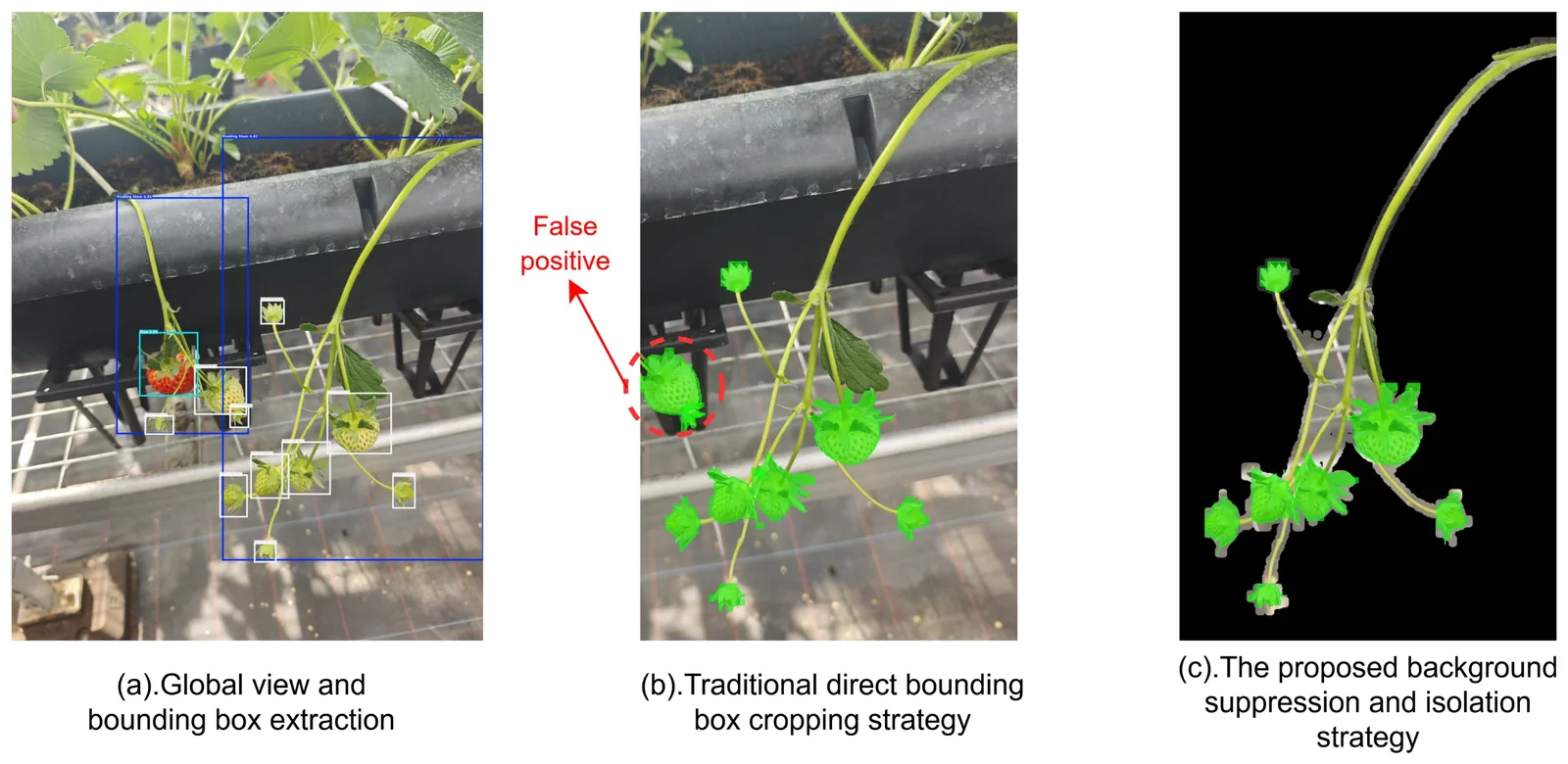
Comparison of local fine-grained segmentation effects between traditional bounding box cropping and the proposed background suppression strategy.

Due to the dense canopy characteristics of strawberry plants, spatial overlapping among adjacent clusters frequently occurs, making conventional vision models prone to feature confusion and erroneous cross-cluster instance associations. To address this challenge, a localized background suppression and isolation strategy was proposed. As illustrated in Figure 12, the effectiveness of the proposed strategy in mitigating surrounding background interference and improving local fine-grained segmentation accuracy is demonstrated.

First, the global segmentation network is deployed to perform coarse localization of the target strawberry clusters (Figure 12a). If a conventional rectangular bounding-box cropping strategy is directly applied (Figure 12b), the background regions within the bounding box inevitably encompass surrounding foliage and neighboring fruit clusters. Consequently, considerable false positive detections may be introduced, as indicated by the red dashed circles in the figure. In contrast, the proposed strategy (Figure 12c) performs target-oriented cropping by exclusively retaining the target regions while replacing non-target background pixels with zero values. This operation effectively isolates irrelevant visual interference and suppresses background clutter, enabling more accurate topological associations between strawberries and their supporting peduncles. To comprehensively quantify the effectiveness of the proposed strategy and eliminate bias from a single dataset partition, evaluations were conducted on the complete independent test set comprising 160 images. Three models trained with distinct random seeds were utilized to perform repeated experiments, and final evaluation metrics were reported as the mean ± standard deviation across the three independent trials.

A detailed quantitative comparison among the conventional bounding-box (Conventional BBox) cropping, the non-dilated mask cropping, and the proposed background suppression strategy is presented in Table 8. The experimental results indicate that the proposed strategy achieved optimal performance across all primary evaluation metrics. The detection precision (${\mathrm{Precision}}_{\mathrm{box}}@0.5$) and mask mean average precision (${\mathrm{mAP}}_{\mathrm{mask}}@0.5$) reached 81.30±1.56% and 79.09±1.37%, respectively. Furthermore, the final fruit–peduncle topological counting accuracy (${\mathrm{Acc}}_{\mathrm{topo}}$) was improved to 76.99±1.23%, demonstrating that the proposed dilated mask strategy effectively preserves the complete morphological features of target clusters while preventing false negatives caused by excessive cropping.

Regarding false positive suppression, the conventional rectangular cropping strategy introduced substantial background clutter into the secondary network, resulting in an average of 1.53±0.28 false positives (FPs) per image. In comparison, the proposed strategy substantially reduced the average FP metric to 0.23±0.02 per image by suppressing background regions to zero intensity, thereby effectively filtering out irrelevant environmental noise.

**Figure 13:**
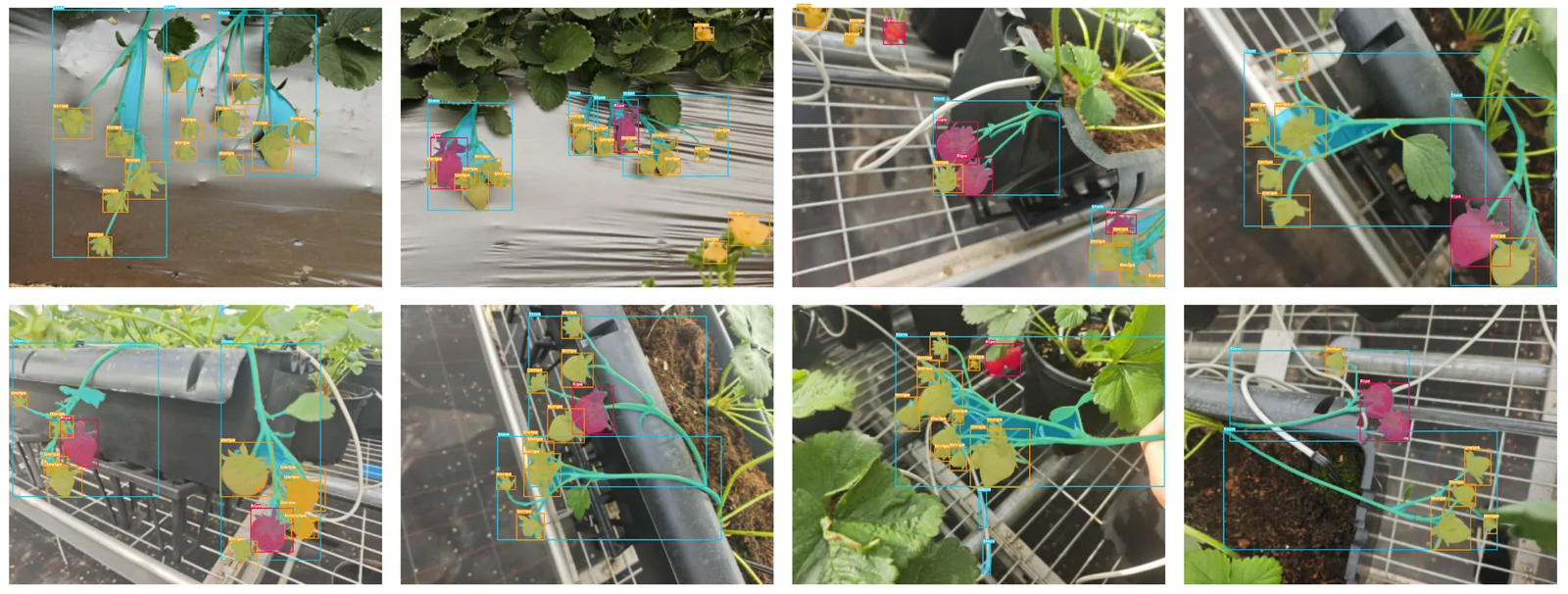
Display of segmentation results for strawberries and fruit stems.

Furthermore, the inherent limitation of the non-dilated mask strategy was also confirmed. Although this strategy achieved the lowest false positive rate (0.12±0.03 FPs per image), its bounding-box precision degraded noticeably to 76.84±1.42%. This performance drop occurred because essential boundary pixels of target organs were truncated during tight contour cropping, leading to increased missed detections. Overall, the proposed strategy achieves a favorable balance between background interference suppression and the structural preservation of target-specific visual features. Representative qualitative instance segmentation results for strawberries and fruit stems are displayed in Figure 13.

#### 3.3.2. Ablation Study Analysis

To rigorously evaluate the contribution of each proposed component to the overall performance of the automated fruit-thinning decision framework and eliminate the influence of random weight initialization, ablation experiments were conducted using YOLOv11n as the baseline architecture. Specifically, all ablation variants were independently trained across three distinct random seeds (42, 1024, and 2026). The detailed evaluation results, reported as the mean ± standard deviation, are summarized in Table 9. As shown in Table 9, the vanilla YOLOv11n baseline achieved a precision, recall, and F1-score of 74.53±2.31%, 63.92±1.20%, and 68.79±0.29%, respectively, with an inference speed of 81 FPS. During subsequent experiments, the proposed architectural modules were progressively integrated into the baseline, and consistent performance improvements were observed.

First, the customized loss function (FTL with dynamic re-weighting) was introduced into the baseline network. This strategy increased the average recall by 3.59 percentage points, reaching 67.51±1.62%, while the F1-score was improved to 70.17±0.29%. These improvements demonstrate that the customized loss formulation effectively alleviates the severe class imbalance inherent in agricultural greenhouse scenarios. Specifically, the strawberry fruit and peduncle dataset exhibits an extreme long-tail distribution across categories, making conventional models prone to omitting minority-class targets. The optimized loss function reinforces gradient feedback from hard samples and minority categories, thereby substantially reducing missed detections caused by skewed data distributions.

Subsequently, the Structural Re-parameterized Convolution (RepConv) module was incorporated into the backbone network. While maintaining comparable segmentation accuracy with an F1-score of 70.13±0.49%, this module primarily functioned as a computational acceleration component. The inference throughput was considerably enhanced, achieving a peak speed of 95 FPS. This performance gain confirms that structural re-parameterization effectively executes operator fusion and simplifies the inference computational graph during deployment, thereby significantly boosting hardware execution efficiency.

Finally, the Coordinate Attention (CoordAtt) mechanism was embedded into the neck feature fusion network. The introduction of this module further boosted the overall detection performance. Specifically, the precision increased to 78.00±1.88%, the recall improved to 70.14±0.78%, and the final F1-score reached a peak value of 73.85±0.56%. Although the fine-grained spatial coordinate modeling introduced a slight computational overhead—causing the inference speed to adjust from 95 FPS to 93 FPS—the final model still substantially outperformed the original baseline in inference efficiency.

Overall, the final integrated model achieved a favorable trade-off between segmentation accuracy and inference speed. By progressively suppressing missed detections, accelerating feature propagation, and strengthening spatial coordinate awareness, the proposed architecture exhibited superior robustness and lightweight characteristics. Consequently, it demonstrates strong suitability for high-accuracy, real-time edge deployment on automated strawberry-thinning robotic platforms.

#### 3.3.3. Comparison of Instance Segmentation Performance with Different Models

To verify the effectiveness of the proposed model, comprehensive comparisons were conducted with classical instance segmentation models, including Mask R-CNN and SOLOv2, as well as representative lightweight YOLO-series networks. As presented in Table 10, although Mask R-CNN and SOLOv2 achieved relatively high detection accuracy, with mAP@0.5 values of 81.34% and 80.27%, respectively, their massive parameter counts and low inference speeds (below 15 FPS) significantly limit their practical deployment on resource-constrained platforms.

Although lightweight YOLO-based models achieved substantially higher inference speeds (75 FPS or above), this throughput improvement was accompanied by a noticeable decrease in detection accuracy. For example, the F1-score of YOLOv8n was only 61.95%.

As shown in Table 10, the proposed RCA-YOLO achieved a favorable trade-off between model lightweightness and detection accuracy. To ensure the reliability and robustness of the evaluation, it should be noted that the reported performance metrics for RCA-YOLO represent the average values obtained from multiple training runs initialized with different random seeds. With a compact parameter size of 2.77 M and computational complexity of 10.2 G FLOPs, the proposed model achieved an mAP@0.5 of 75.73% and an F1-score of 73.85%, consistently outperforming the baseline lightweight YOLO models. Furthermore, RCA-YOLO achieved an inference speed of 92 FPS, satisfying the real-time detection requirements of agricultural robotic operations. These results demonstrate the effectiveness of the proposed structural modifications in enhancing the feature representation capability while maintaining high inference efficiency.

It is worth noting that the model’s recall of 70.14% reflects a practical trade-off between inherent target physical characteristics and the strict constraints of lightweight edge computing. Physically, undetected targets predominantly represent challenging hard samples: slender peduncles that share green color textures with background foliage and are heavily occluded within dense fruit clusters. Architecturally, to satisfy the low-latency constraints of agricultural robots, the proposed network maintains an extremely low parameter overhead. While heavyweight models achieve higher recall on these occluded samples through deeper feature extraction, they fail to meet the real-time throughput demands of edge platforms. Therefore, a compromised recall for severely occluded targets represents an acceptable and practical necessity to ensure high-speed, robust edge execution.

**Figure 14:**
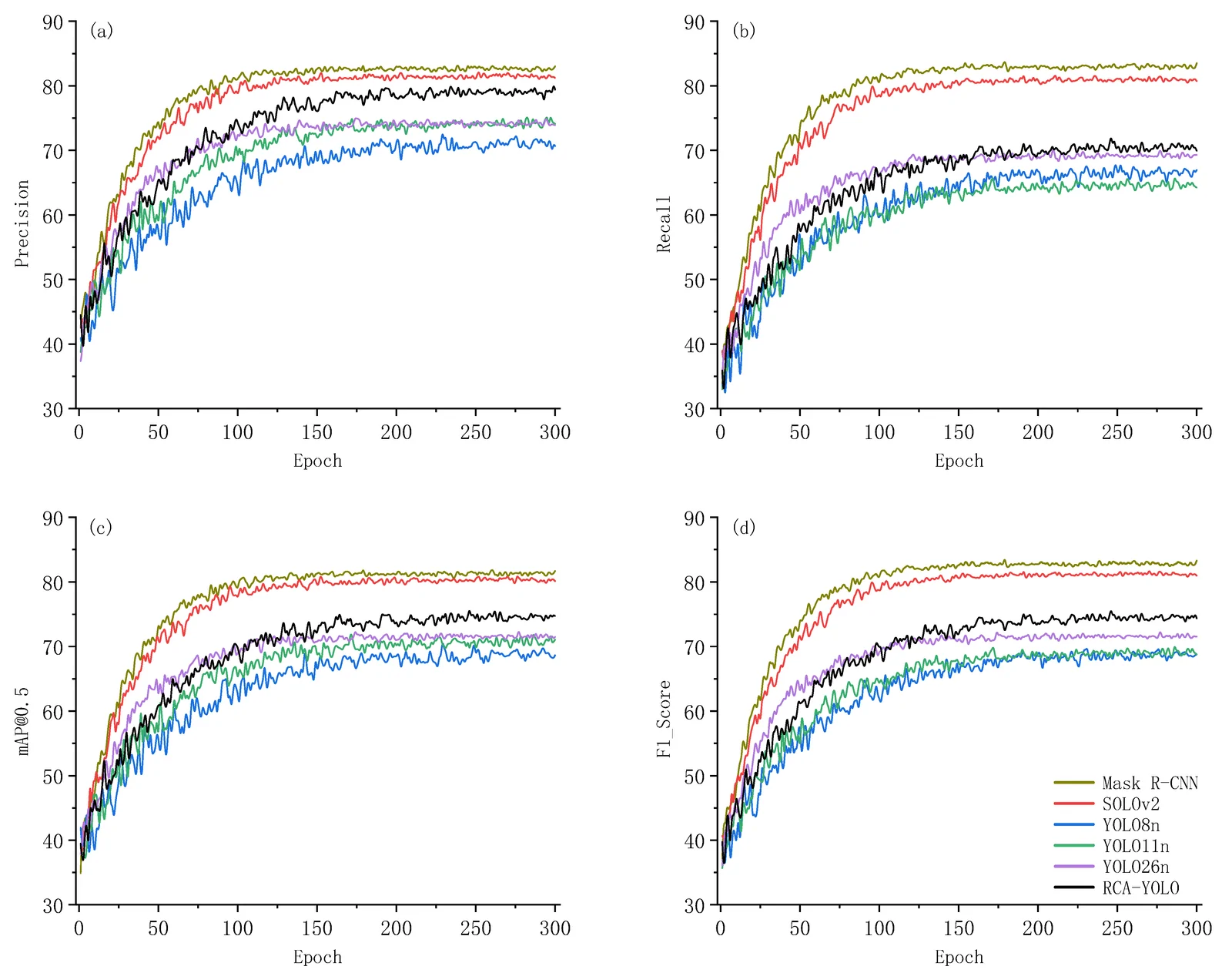
Comparative training curves of the evaluated models over 300 epochs: (**a**) precision; (**b**) recall; (**c**) mAP@0.5; and (**d**) F1_Score.

As illustrated in Figure 14, the dynamic training trajectories of the proposed RCA-YOLO and the five comparative models (Mask R-CNN, SOLOv2, YOLOv8n, YOLOv11n, and YOLOv26n) were monitored over 300 epochs.

All models exhibited rapid performance improvements during the initial 50 epochs and gradually converged after approximately 150 epochs. Heavyweight models (Mask R-CNN and SOLOv2), benefiting from deeper feature extraction backbones and larger parameter capacities, established higher performance upper bounds across all evaluation metrics. In contrast, standard lightweight baselines (YOLOv8n, YOLOv11n, and YOLOv26n) exhibited comparatively lower convergence levels, indicating limited feature representation capacity in dense agricultural scenarios.

Notably, throughout the training process, the proposed RCA-YOLO consistently maintained higher accuracy than the other lightweight YOLO variants in terms of precision, recall, mAP@0.5, and F1-score. While preserving a compact architectural footprint, the learning trajectory of RCA-YOLO effectively narrowed the performance gap between lightweight networks and heavyweight models. This further confirms that the integrated architectural enhancements effectively strengthen the feature learning capability and multi-scale detection accuracy.

#### 3.3.4. Practical Edge Deployment

**Figure 15:**
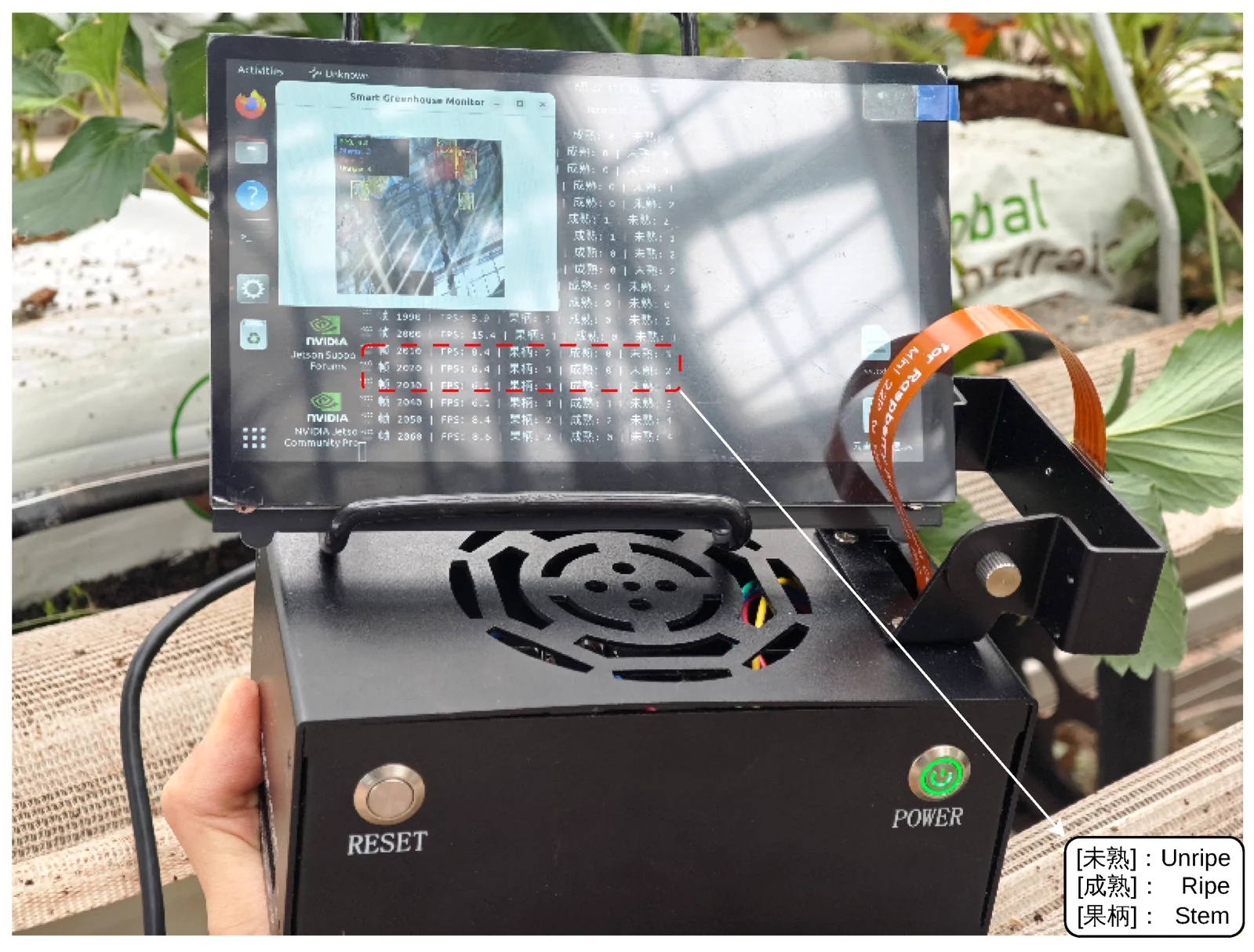
Field deployment test of edge computing devices in a greenhouse.

To validate the practical feasibility of the proposed hierarchical perception system for actual agricultural robotic operations, the developed algorithm was transferred from a high-performance server environment to a resource-constrained embedded edge computing platform (Jetson Orin Nano Super) for deployment evaluation. The edge deployment experiment was designed to assess the model’s accuracy preservation capability after half-precision quantization (FP16) and evaluate the end-to-end real-time throughput of the proposed three-stage hierarchical processing pipeline on physical embedded hardware. The experimental setup and on-site deployment test of the embedded hardware in the greenhouse environment are presented in Figure 15.

During edge deployment, directly executing the original PyTorch dynamic graph model (.pt format) incurred substantial memory consumption and computational latency. To overcome the computational constraints of embedded hardware, the NVIDIA TensorRT inference optimization engine was introduced. The original model was recompiled and serialized into the .engine format, enabling acceleration through FP16 half-precision floating-point computation. To evaluate the potential impact of FP16 quantization on feature representation capability, quantitative accuracy comparisons were conducted on the same test set between the server-side model (FP32 precision) and the edge-side model (FP16 precision). The detailed evaluation results are summarized in Table 11.

As shown in Table 11, only negligible degradation was observed in the core evaluation metrics after TensorRT optimization and half-precision quantization. To ensure the reliability of the evaluation, all reported data represent the average values obtained from three models trained with distinct random seeds. Specifically, the precision decreased by only 0.07%, while the reductions in F1-score and mAP@0.5 were maintained within a minimal range of 0.17–0.42%. These results indicate that the adopted YOLOv11n-seg architecture exhibits strong robustness in extracting features from densely clustered strawberry canopies. FP16 quantization effectively reduced memory bandwidth requirements; although the slight decrease in mAP@0.5 (from 75.73% to 75.31%) reflects a marginal precision trade-off rather than a strictly lossless conversion, this minor variance is practically negligible, thereby satisfying the operational accuracy requirements for automated agricultural applications.

After confirming the edge-side perception accuracy, the end-to-end processing speed (FPS) served as a critical engineering indicator determining whether the strawberry-thinning robotic system could operate smoothly. In the proposed coarse-to-fine hierarchical segmentation framework, each image frame undergoes three sequential stages: global coarse inference, CPU-based mask morphology and background suppression processing, and local high-resolution ROI fine inference. Therefore, the actual computational cost cannot be directly estimated by simply accumulating the inference time of individual network forward passes. To accurately quantify the computational overhead, an end-to-end latency decomposition and frame-rate stress test were conducted on the edge platform, as summarized in Table 12.

As indicated in Table 12, when TensorRT was deployed solely for a single-pass standard YOLO instance segmentation inference, the maximum achievable throughput reached 34.80 FPS (28.73 ms per frame). However, under densely clustered and overlapping strawberry canopies, single-pass inference cannot effectively resolve spatial topological ambiguities. After introducing the proposed three-stage hierarchical segmentation strategy, the complete algorithmic pipeline—encompassing dual network inferences alongside intermediate OpenCV-based mask dilation and ROI extraction—required 61.47 ms per frame. Accounting for the additional 2.70 ms data transfer overhead induced by CSI camera acquisition and memory copying, the total end-to-end latency reached 64.17 ms, stabilizing the final end-to-end system throughput at 15.58 FPS.

**Figure 16:**
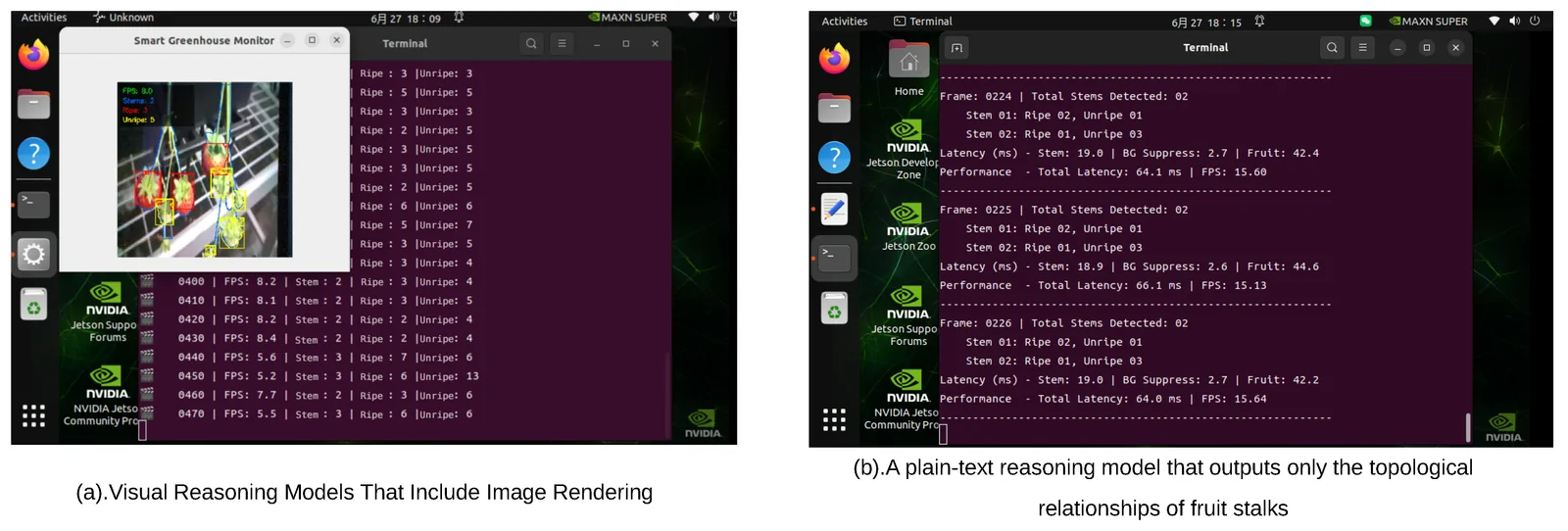
Testing the deployment of dual-mode inference on edge devices.

Although the hierarchical strategy reduced the absolute frame rate from 34.80 FPS to 15.58 FPS, this performance trade-off provides substantial engineering value for field robotic applications. First, automated fruit-thinning robotic systems typically operate under an eye-to-hand configuration or a sequential “move–stop–observe–actuate” operational routine, where the physical actuation latency of robotic arms and end-effectors is generally at the scale of hundreds of milliseconds. The proposed system achieves a single-frame visual decision latency of approximately 64.17 ms (15.58 FPS), which is considerably faster than the mechanical actuation cycle. Consequently, the visual perception latency is effectively prevented from becoming the operational bottleneck within the overall control loop. The practical deployment and real-time execution of the dual-mode inference on edge devices are illustrated in Figure 16.

Second, by introducing an incremental computational overhead of approximately 32.7 ms, the hierarchical strategy effectively suppresses extraneous background clutter at the pixel level and establishes robust topological associations between target fruits and their supporting peduncles. Consequently, the risk of destructive physical cutting actions caused by visual misjudgment is significantly mitigated at the perception stage. Therefore, the proposed edge deployment scheme not only satisfies real-time processing constraints under limited embedded computing resources, but also considerably enhances decision reliability and operational safety.

## 4. Discussion

To address the challenges associated with complex cultivation environments and the establishment of target topological relationships in intelligent strawberry fruit thinning, the proposed hierarchical perception framework demonstrates distinct advantages over existing mainstream vision models in both fruit–peduncle association establishment and lightweight edge deployment.

Current agricultural vision algorithms are largely restricted to the isolated localization or instance segmentation of strawberry fruits and stems. For example, the E-YOLACT model derived from the YOLACT framework achieved a Mask-mAP of 76.6% and an inference speed of 34.8 FPS in complex cultivation environments [28]. However, the outputs of such models remain confined to independent object bounding boxes and segmentation masks. Due to the absence of spatial dependency reasoning mechanisms, these methods cannot establish the topological association between specific fruits and their supporting peduncles at the data level, making them insufficient to satisfy the stringent hierarchical criteria required for automated fruit thinning.

To establish such topological associations, another category of mainstream approaches introduces human pose estimation techniques for keypoint detection. For instance, Xiong et al. employed a deep learning vision system to recognize strawberries in unstructured environments, estimating optimal harvesting positions by extracting spatial geometries and computing relative distances between fruits and the robotic gripper [29]. Similarly, the DHN-YOLO model proposed by Hao et al. in 2025 performed keypoint detection based on the YOLO11-pose framework [30]. However, in high-density strawberry clusters where multiple fruits and peduncles overlap due to perspective projection, the nearest keypoint may belong to an adjacent, physically unrelated fruit, leading to erroneous topological binding.

In contrast, the proposed coarse-to-fine three-stage perception pipeline overcomes the limitations of conventional approaches that rely on heuristic geometric rules. Through localized cropping combined with background suppression, visual interference caused by overlapping stems and background foliage is physically eliminated. Under these isolated conditions, the generated polygon masks enable reliable logical associations between fruits and their corresponding peduncles. Consequently, the average false positive (FP) detections per image were reduced from 1.53 to 0.23, resolving the critical challenge of accurately determining fruit–peduncle topological relationships in automated thinning scenarios.

To resolve feature ambiguity induced by illumination fluctuations and shadows on slender peduncles (with diameters of only 1–2 mm), several recent studies have explored multimodal deep fusion approaches. For example, Tamrakar et al. proposed the DF-Mask R-CNN architecture in 2025, which integrates dense depth maps generated by a monocular ZoeDepth estimator with RGB images to enhance feature perception [31]. However, this framework relies on a computationally intensive ResNet101 backbone comprising tens of millions of parameters. Moreover, monocular depth estimation is prone to visual artifacts and depth discontinuities along slender peduncle boundaries. The substantial memory footprint and computational overhead of such multimodal networks render real-time execution on resource-constrained edge platforms impractical.

While heavyweight models such as Mask R-CNN and SOLOv2 achieve higher mAP scores (exceeding 80%), it is crucial to objectively assess this performance within the operational context of agricultural edge deployment. The superior accuracy of conventional models comes at the expense of massive parameter volumes and high FLOPs. Consequently, when deployed on resource-constrained embedded devices, these models suffer from excessive latency and high power consumption, rendering them incapable of meeting the real-time continuous operation requirements of agricultural robots. In contrast, the proposed lightweight pipeline prioritizes deployment feasibility over the theoretical maximum accuracy. The model achieves an mAP@0.5 of 75.73%, which, when coupled with the drastically suppressed false positive rate of the three-stage framework, provides sufficient precision and operational safety for automated thinning tasks. Therefore, this trade-off is not merely a compromise, but a deliberate optimization that shifts the design focus from maximizing workstation GPU metrics to ensuring real-time, low-power, and robust execution on agricultural edge nodes.

Furthermore, the proposed approach utilizes exclusively monocular RGB images and overcomes the challenge of extracting slender morphological features under extreme class imbalance by introducing Structural Re-parameterized Convolution (RepConv) and Focal Tversky Loss. Without increasing network depth, the model size was condensed to a compact 2.77 M parameters. Following TensorRT-based FP16 acceleration on the Jetson Orin Nano platform, the model achieved near-lossless precision transfer, exhibiting only a 0.42% decrease in mAP@0.5. Concurrently, the end-to-end processing throughput reached 15.58 FPS with an algorithmic processing latency of 61.47 ms per frame. These experimental outcomes demonstrate that, under constrained embedded computational resources, carefully engineered algorithmic pipelines provide a practical and cost-effective alternative to computationally demanding large-scale models.

**Figure 17:**
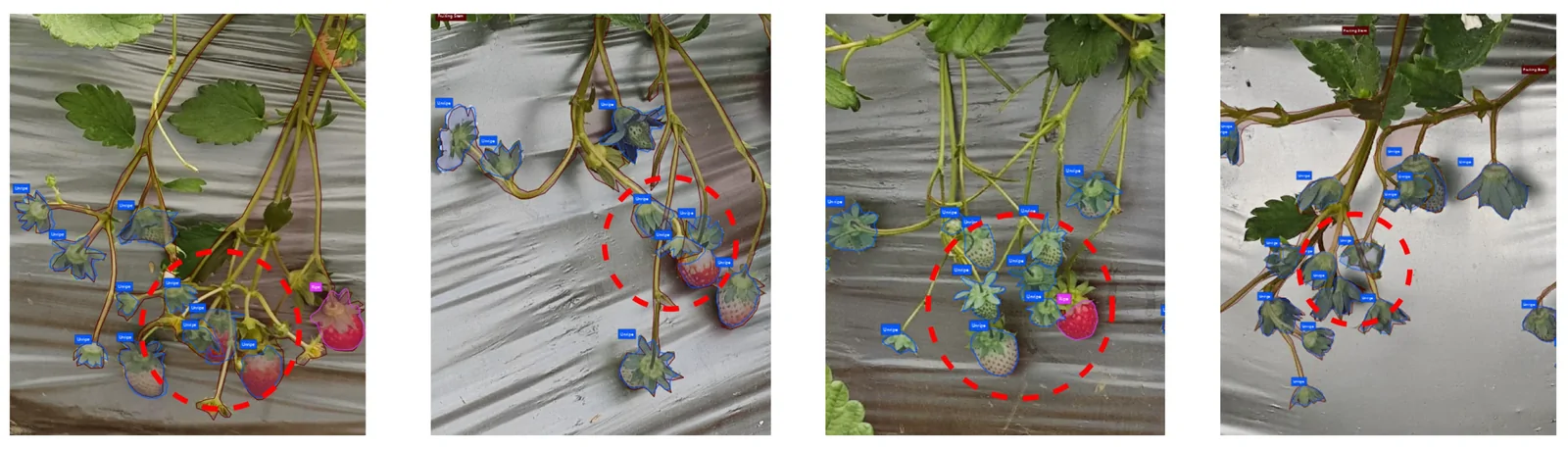
Examples of incorrect fruit attribution in densely intertwined scenes.

Although the proposed method operates efficiently with minimal computational requirements in most fruit-thinning scenarios, operational limitations remain when extremely dense strawberry clusters are encountered. As illustrated by the red dashed region in Figure 17, strawberries frequently grow in dense clusters with interwoven stems. When fruits from neighboring peduncles extend into or interlock around the target cluster, severe cross-cluster interference occurs. Representative failure cases of incorrect fruit attribution caused by such cross-cluster interference are shown in Figure 17.

Such spatial interference may degrade the effectiveness of the localized cropping strategy. Within the cropped local sub-image, global contextual semantics are partially reduced, causing the network to rely primarily on localized feature patterns and spatial proximity to determine fruit attribution. If an extraneous fruit is positioned in close proximity to the target peduncle while its own supporting stem is occluded by foliage, the model may misinterpret spatial proximity as belonging to the target stem. This ambiguity can result in adjacent fruits being erroneously assigned to the current peduncle, leading to local over-counting.

Nevertheless, during practical robotic operations, this visual limitation is effectively mitigated through two primary engineering mechanisms. First, a conservative fail-safe strategy is integrated into the decision-making logic: within highly ambiguous overlapping regions, the suppression of false positives is strictly prioritized over the avoidance of false negatives. For instance, a false positive could induce an erroneous robotic cutting action (e.g., mistakenly severing the main peduncle), resulting in irreversible yield loss. Second, static visual occlusions are dynamically compensated for by the continuous movement of the robotic manipulator. Facilitated by the high inference frame rate, dynamic multi-view image sequences are continuously acquired as the robotic arm advances. As the operational viewpoint shifts, static occlusions present in a single frame are naturally eliminated, exposing previously hidden structures to enable successful recognition.

In future research, multi-view spatial fusion and graph convolutional reasoning will be integrated into the framework to resolve severe cross-cluster occlusions and attribution ambiguities. Furthermore, the topological counting outputs will be directly coupled with the automated fruit-thinning decision system. For instance, based on agronomic criteria, if the number of fruits associated with a single peduncle exceeds five, that peduncle is flagged as requiring thinning, and this operational command is transmitted to the downstream robotic control system. This integration will further advance the operational efficiency, robustness, and autonomy of robotic strawberry-thinning systems.

## 5. Conclusions

To address the critical challenges encountered in intelligent strawberry fruit thinning—including complex cultivation environments, ambiguous fruit–peduncle topological relationships, and constrained computational resources on edge devices—a lightweight hierarchical instance segmentation framework tailored for edge deployment was developed. The primary conclusions are summarized as follows.

A coarse-to-fine three-stage perception pipeline was proposed to eliminate topological association errors induced by dense canopy occlusions. By sequentially executing global localization, localized cropping with background suppression, and fine-grained secondary inference, visual interference from complex environmental backgrounds was effectively isolated. This strategy substantially reduces per-image false positive detections and ensures accurate fruit–peduncle topological binding, fundamentally mitigating the operational risk of erroneous robotic cutting actions.

Furthermore, a dedicated benchmark dataset focusing on strawberry organ-level topology was constructed, providing fine-grained polygon mask annotations for slender peduncles to bridge existing public data gaps. Built upon this foundation, the proposed RCA-YOLO model integrates Structural Re-parameterized Convolution (RepConv), Coordinate Attention (CoordAtt), and Focal Tversky Loss. This architectural design achieves a favorable trade-off between minimizing model complexity and maximizing feature extraction capability for slender, occluded peduncles.

Efficient real-time deployment was successfully demonstrated on an embedded edge computing platform. By coupling TensorRT optimization with FP16 quantization, near-lossless precision migration and high-throughput inference were attained with exceptional engineering cost-effectiveness, thereby satisfying the real-time response constraints of automated field robotic operations. By accurately identifying fruit–peduncle topological associations, this framework establishes a reliable perception baseline for automated thinning decisions. The precise execution of scientific thinning effectively optimizes nutritional distribution and regulates fruit spatial load, directly enhancing the sweetness, palatability, and single-fruit weight of strawberries. Furthermore, dense-cluster disease infection risks are significantly reduced, minimizing pesticide reliance and safeguarding the sustainable production of high-quality food.

Although a reliable topological perception pipeline was demonstrated in this study, the current experimental data remain confined to specific cultivation facilities. Future research will focus on expanding the dataset across diverse geographic regions, climate conditions, and strawberry cultivars to comprehensively evaluate and further strengthen the model’s cross-domain generalization capabilities.

## Figures and Tables

**Table 1:** Parameter settings for strawberry image data collection.

Parameter	Specification	Purpose
Collection Period	December 2025–March 2026	Covers diverse fruit growth stages
Original Resolution	4094 × 3072 pixels	Ensures clarity of thin peduncles and overlaps
Sensor Size	1/1.31 inch	Increases signal-to-noise ratio
Aperture	f/1.6	Ensures sufficient light intake in shaded foliage areas
Pixel Pitch	1.2 μm/2.4 μm	Resolves fine peduncle structures
Autofocus	Octa-PD	Maintains sharp focus on targets
Color Space	RGB (3-channel)	Preserves phenotypic color features
Image Format	JPG/JPEG	Facilitates standard data augmentation
Shooting Distance	30–50 cm	Replicates authentic field operation distance

**Table 2:** Definitions and instance statistics of annotation categories.

Category Name	Quantity	Definition
Fruit Stem	2238	The complete unit of a peduncle and its attached fruit.
Ripe	4094	Red area > 75% of the visible fruit surface
Unripe	10,926	Red area < 75% of the visible fruit surface, or only the calyx visible

**Table 3:** Partitioning and statistics of the strawberry instance segmentation dataset.

Subset	Number of Images	Proportion (%)	Primary Purpose
Training Set	812	71.80%	Model training (including augmented samples)
Validation Set	159	14.06%	Hyperparameter tuning and model selection
Testing Set	160	14.14%	Independent performance evaluation
Total	1131	100.00%	Whole-image level split (≈7:1.5:1.5)

**Table 4:** Symbol definitions for the fused mask and initial area calculations.

Symbol	Description
$M_{\mathrm{base}}\left(x,y\right)$	Pixel value of the fused base mask at (x,y)
*N*	Total number of detected instances (stems and fruits)
⋁	Pixel-wise logical OR (union) operation
$S_{i}\left(x,y\right)$	Pixel value of the *i*-th binary instance mask at (x,y)
W,H	Width and height of the input image
$A_{\mathrm{orig}}$	Total foreground pixel area of the fused mask
∑∑	Summation over the entire 2D image space

**Table 5:** Performance comparison of different mask expansion rates and crop buffer ratios on the validation set.

Parameter	Value (%)	mAP@0.5 (%)	Recall (%)
Mask Expansion	10	72.15	65.30
	15	73.80	68.10
	**20 (Ours)**	**74.68**	**70.58**
	25	73.10	72.40
	30	71.45	74.10
Crop Buffer	0	71.80	68.20
	**2 (Ours)**	**74.68**	**70.58**
	5	73.50	71.20
	8	72.20	71.50

Note: Values in bold indicate the optimal parameters and corresponding superior performances achieved by our proposed method.

**Table 6:** Configuration of experimental hardware and software environment.

Environment Type	Item	Configuration Parameters/Version
Hardware Environment	CPU	Intel Xeon Platinum 8368Q @ 2.60 GHz
	GPU	NVIDIA GeForce RTX 5090 (32 GB VRAM)
	Memory (RAM)	128 GB
Software Environment	Operating System	Windows 10 Professional (64-bit)
	Programming Language	Python 3.11.15
	Deep Learning Framework	PyTorch 2.12.0
	Compute Architecture	CUDA 12.9

**Table 7:** Hyperparameter Configuration for Network Training.

Hyperparameter	Value/Configuration	Purpose & Remarks
Input Image Size	640 × 640 pixels	Unifies resolution to balance speed and feature extraction
Batch Size	32	Number of samples processed per gradient update
Total Epochs	300	Adapts to RepConv and CoordAtt for stable updates
Optimizer	AdamW	Facilitates standard data augmentation
Initial Learning Rate	0.001	Baseline learning rate for the initial training phase
Warm-up Epochs	5 epochs	Gradually scales up learning rate to prevent early gradient explosion
Loss Weight Ratio	5.0:2.5:1.5	Bounding Box Regression Loss:Classification Loss:DFL Loss
Label Smoothing	0.2	Suppresses network overfitting in complex, occluded scenarios

**Table 8:** Comparison of detection and classification performance between traditional rectangular-box segmentation and the strategy proposed in this paper.

Method	${\mathrm{Precision}}_{\mathrm{box}}@0.5$	${\mathrm{mAP}}_{\mathrm{mask}}@0.5$	${\mathrm{Acc}}_{\mathrm{topo}}$	Average FP per Image
Conventional BBox	80.34 ± 1.54%	75.03 ± 1.93%	70.38 ± 2.22%	1.53 ± 0.28
Non-dilated Mask	76.84 ± 1.42%	75.14 ± 1.54	73.52 ± 1.64%	0.12 ± 0.03
**Ours**	**81.30 ± 1.56%**	**79.09 ± 1.37%**	**76.99 ± 1.23%**	**0.23 ± 0.02**

Note: In the table, Conventional BBox refers to the conventional bounding-box cropping strategy; Non-dilated Mask refers to the non-dilated mask cropping strategy; and Ours refers to the segmentation strategy proposed in this study. Values in bold indicate the superior performance achieved by our proposed method.

**Table 9:** Statistics of evaluation metrics for ablation experiments.

Baseline	Loss Function	RepConv	Coordinate	Precision (%)	Recall (%)	F1-Score (%)	FPS
YOLOv11n				74.53 ± 2.31	63.92 ± 1.20	68.79 ± 0.29	81
	✓			73.11 ± 2.10	67.51 ± 1.62	70.17 ± 0.29	79
	✓	✓		75.63 ± 2.20	65.42 ± 0.92	70.13 ± 0.49	95
	✓	✓	✓	78.00 ± 1.88	70.14 ± 0.78	73.85 ± 0.56	93

Note: ✓ indicates the inclusion of the corresponding component or strategy.

**Table 10:** Statistics of experimental results on comparative evaluation metrics for different models.

Model	Precision	Recall	mAP@0.5	F1-Score	Params (M)	FLOPs (G)	FPS
Mask R-CNN	82.69%	83.00%	81.34%	82.84%	43.93	88.92	14
SOLOv2	81.51%	81.00%	80.27%	81.25%	46.18	54.77	13
YOLOv8n	71.37%	66.93%	69.08%	61.95%	3.26	11.5	114
YOLOv11n	74.38%	64.76%	70.92%	66.08%	2.84	9.7	85
YOLOv26n	74.14%	69.22%	71.60%	64.19%	3.05	10.2	75
**RCA-YOLO**	**78.00%**	**70.14%**	**75.73%**	**73.85%**	**2.77**	**10.2**	**92**

Note: Values in bold indicate the superior performance and optimal trade-off achieved by our proposed RCA-YOLO model.

**Table 11:** Comparison of instance segmentation accuracy between server and edge computing platforms.

Deployment Platform	Precision	Recall	mAP@0.5	F1-Score
High-Performance Server	78.00%	70.14%	75.73%	73.85%
Jetson Orin Nano Super	77.93%	69.87%	75.31%	73.68%

**Table 12:** Comparison of inference duration between single-step inference at the edge and a three-stage hierarchical system.

Inference Mode	Processing Time per Frame (ms)	Data Transfer Overhead (ms)	End-to-End FPS
Single-Pass Standard Inference	28.73	≈0	34.80
Proposed Three-Stage Hierarchical Segmentation	61.47	≈2.70	15.58

## Data Availability

The original contributions presented in this study are included in the article. Further inquiries can be directed to the corresponding authors.
